# Improving the reliability of model-based decision-making estimates in the two-stage decision task with reaction-times and drift-diffusion modeling

**DOI:** 10.1371/journal.pcbi.1006803

**Published:** 2019-02-13

**Authors:** Nitzan Shahar, Tobias U. Hauser, Michael Moutoussis, Rani Moran, Mehdi Keramati, NSPN consortium, Raymond J. Dolan

**Affiliations:** 1 Wellcome Centre for Human Neuroimaging, University College London, London, United Kingdom; 2 Max Planck UCL Centre for Computational Psychiatry and Ageing Research, London, United Kingdom; Harvard University, UNITED STATES

## Abstract

A well-established notion in cognitive neuroscience proposes that multiple brain systems contribute to choice behaviour. These include: (1) a model-free system that uses values cached from the outcome history of alternative actions, and (2) a model-based system that considers action outcomes and the transition structure of the environment. The widespread use of this distinction, across a range of applications, renders it important to index their distinct influences with high reliability. Here we consider the two-stage task, widely considered as a gold standard measure for the contribution of model-based and model-free systems to human choice. We tested the internal/temporal stability of measures from this task, including those estimated via an established computational model, as well as an extended model using drift-diffusion. Drift-diffusion modeling suggested that both choice in the first stage, and RTs in the second stage, are directly affected by a model-based/free trade-off parameter. Both parameter recovery and the stability of model-based estimates were poor but improved substantially when both choice and RT were used (compared to choice only), and when more trials (than conventionally used in research practice) were included in our analysis. The findings have implications for interpretation of past and future studies based on the use of the two-stage task, as well as for characterising the contribution of model-based processes to choice behaviour.

## Author summary

In this paper, we report a reliability analysis for the estimation of “model basedness”—a psychological construct that informs a wealth of studies in animal, human and clinical research. We consider an exemplar paradigm, the two-step task, widely used in the recent literature. We report low reliability for model-agnostic model-based estimates, as well as computational model parameter estimates. We suggest how a model-based/free trade-off might affect reaction-time variability in this task, and go on to suggest use of model parameter estimates based on a combination of choice and RT. Finally, we demonstrate that combining choice and RT estimates improves both model-agnostic and algorithmic model-based estimates.

## Introduction

Animal and human decision-making research suggests that when an agent deliberates on a course of action more than one control system contributes to choice. A dominant idea invokes a contribution of a model-free and a more sophisticated goal-directed model-based system. Both influence the choice process but their relative influence is assumed to vary between individuals and conditions [1]. Theoretical questions regarding human planning [2], reasoning [3], development [4], voluntary-control [5], learning under uncertainty [6], motor control [7], and deployment of attentional resources [8], all invoke this dichotomy. Human neuroimaging studies suggest these two systems rely on distinct neural substrates [9–11]. Additionally, various hypotheses regarding the clinical relevance of this distinction have been put forward [12], with relative deficits in a model-based system suggested as underpinning clinical conditions such as compulsivity [13,14], substance-use [15], and obesity [16]. Even moral judgments, especially of a deontological nature, are suggested to include a model-free and model-based contributions [17].

The contribution of model-free and model-based systems is often probed via a multi-step decision tree task, wherein participants are asked to make state selections (navigate their way in an artificial maze) to attain goal rewards [18]. In this paradigm, a habitual model-free learner is assumed to select actions based on a reward history alone, without considering task structure. In contrast, to make better choices, a model-based learner is assumed to avail of a cognitive map that takes account of transition structure. While in some cases these two systems can lead to the same action, the task also generates a scenario where these two systems lead to different actions, enabling measurement of their relative contribution to the decision-making process [18–20].

**Fig 1:**
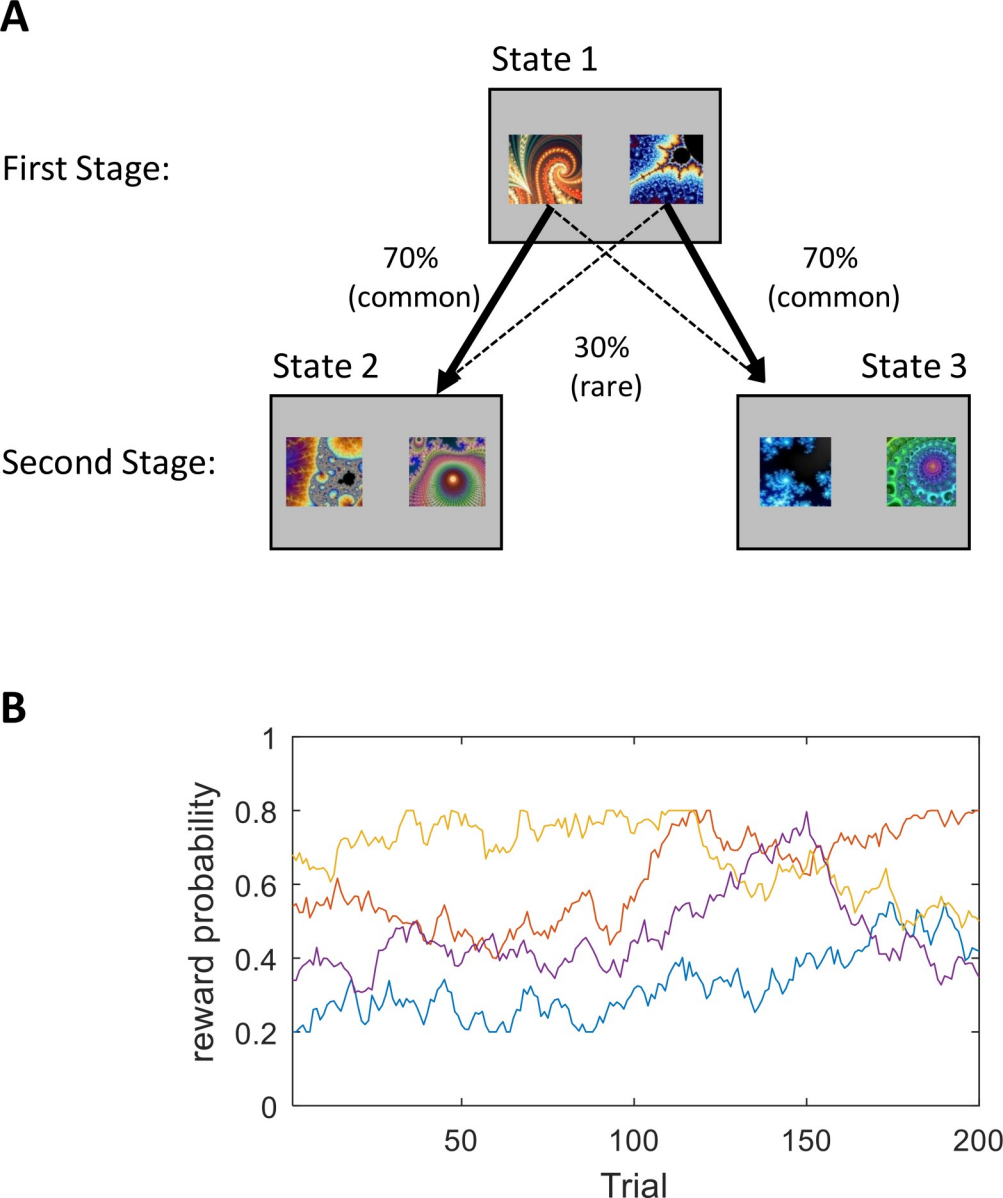
A schematic of the two-stage task (panel A) and an example of a random walk used to generate the true expected value for each of the four bandits at the task second-stage (panel B). At the first-stage participants choose between two options (represented by abstract fractal images) that determined the presentation of the second-stage via fixed transition probabilities of 70% (‘common’) or 30% (‘rare’). At the second-stage, participants again choose between two bandits that led to receipt of reward (£0 or £1 play pounds). Note the second-stage included two pairs of bandits where the composition of each pair was fixed, but where the value of each bandit drifted slowly and independently. More specifically the reward associated with the second-stage bandits were subjected to random walks and thus had to be constantly learned by participants.

In the two-stage decision task participants navigate from a first to a second stage to gain rewards (see Fig 1). The second stage usually entails four bandits and participants make first stage choices that probabilistically determine which bandits are available at the second stage (see Fig 1). First-stage choices provide descriptive measures as well as model parameters that quantify the contribution of model-free and model-based systems [21]. Thus, when making a first-stage choice, a model-free system considers whether an action led previously to a reward. In contrast, a model-based system also considers the transition probabilities. For example, a model-based learner might select a specific action that was not rewarded on the last trial, based on an inference form task structure that it will more reliably lead to a more rewarding second-stage state.

This two-stage decision task is considered a gold-standard for measuring model-based/model-free contributions to choice behaviour across computational [21], neuroimaging [2], behavioural [22], developmental [4] and clinical studies [13,23]. Variants of the task are also used in animal studies [24]. Despite its widespread use no study has yet provided task reliability estimates. Almost all studies make exclusive use of a metric of choice, but not reaction-time (RT), to derive estimates of model-basedness. However, a decision-making literature indicates RT data is an important source of information [25,26], with a recent study suggesting it might improve parameter identifiability in reinforcement learning paradigms [27]. It remains unclear whether a combination of choice and RT data might improve the reliability of model-based scores.

Here, using the two-stage task we estimated the reliability of model-based estimates by exploiting a large data set which included two distinct testing time points derived from the Neuroscience in Psychiatry Network’s study [28]. We started by describing a widely used computational model [18] that allows quantification of model-based/free trade-off in first-stage choices. While the latter model is designed to predict choice, we extended on this model to determine whether a combination of choice and RT data might improve parameter recovery, as well as improve the reliability of model-based estimates [27]. We demonstrate a relationship between model-parameters and model-agnostic measures; one based on choices at the first stage and used widely in past studies, and the other based on RTs at the second stage that is much less reported in the literature [4,29,30]. We reported internal (within measurement) and temporal (between measurements) stability of model-parameters and model-agnostic scores, separately and combined. Overall, our study allowed estimation of psychometric properties of choice-based model-based estimates and demonstrates how these can be improve upon using RTs.

### Model-parameter estimates for model-based/free trade-off

#### w-parameter I (RL model, choice only)

Daw at el., (2011) [18] reported a reinforcement learning (RL) model that allows quantification of a model-based/free trade-off from first-stage choice behaviour. Here, the value of each first-stage bandit was the sum of two components: (1) model-free value–reflecting the amount of previous reward that followed this bandit selection, and (2) model-based value–reflecting the highest value of the two bandits that is reached by a common/rare transition following this first-stage action. In simple terms, while the model-free component was “keeping tabs” of reward history following the selection of a first-stage action, the model-based component was “looking forward” into the second-stage, and considered the best bandit that a first-stage action was likely to lead to (by means of a common transition). The model first updated model-free Q-values at each trial, which were initialized to zero at the beginning of the experiment, and updated at the end of each trial according to a SARSA reward prediction error algorithm [31,32].

Let a_1_/a_2_ be the actions selected in the first/second stage of the task and reward at trial *t* be *r*_*(t)*_*ϵ*{0,1}. The values of the actions in the second-stage were updated according to:

$${\text{Q}}^{\text{M}\text{F}}{}_{\left(\text{a}2,\text{t}+1\right)}={\text{Q}}^{\text{M}\text{F}}{}_{\left(\text{a}2,\text{t}\right)}+\alpha \left({\text{r}}_{\left(\text{t}\right)}-{\text{Q}}^{\text{M}\text{F}}{}_{\left(\text{a}2,\text{t}\right)}\right)$$ (1)

where α was a learning rate (free parameter) and (r_(t)_-Q^MF^_(a2,t)_) represented a reward prediction error. The model-free values of first-stage actions were updated using both the value of the second-stage action, and reward prediction error of the second-stage action:

$${\text{Q}}^{\text{M}\text{F}}{}_{\left(\text{a}1,\text{t}+1\right)}={\text{Q}}^{\text{M}\text{F}}{}_{\left(\text{a}1,\text{t}\right)}+\alpha \;\left({\text{Q}}^{\text{M}\text{F}}{}_{\left(\text{a}2,\text{t}\right)}-{\text{Q}}^{\text{M}\text{F}}{}_{\left(\text{a}1,\text{t}\right)}\right)+\alpha \;\lambda \left({\text{r}}_{\left(\text{t}\right)}-{\text{Q}}^{\text{M}\text{F}}{}_{\left(\text{a}2,\text{t}\right)}\right)$$ (2)

where λ was the eligibility trace (free parameter) capturing the effect of the second-stage prediction error on the first-stage action value.

The model-based learning strategy incorporated the empirical transition probabilities and second-stage Q^MF^ values to estimate the value of first-stage actions according to:

$${\text{Q}}^{\text{M}\text{B}}{}_{\left(\text{a}1,\text{t}\right)}=\text{P}\left({\text{s}}_{2}|{\text{a}}_{1}\right)*\text{m}\text{a}\text{x}\left({\text{Q}}^{\text{M}\text{F}}{}_{\left(\text{s}2,\text{t}\right)}\right)\;+\;\text{P}\left({\text{s}}_{3}|{\text{a}}_{1}\right)*\text{m}\text{a}\text{x}\left({\text{Q}}^{\text{M}\text{F}}{}_{\left(\text{s}3,\text{t}\right)}\right)$$ (3)

where s_2_/s_3_ represented the two states in the second-stage (see Fig 1), and P(s2|a1)/ P(s3|a1) the transition probability. Finally, a w-parameter quantified model-based vs. model-free trade-off in first-stage actions, allowing the calculation of a net Q-value for each first-stage action:

$${\text{Q}}^{\text{n}\text{e}\text{t}}{}_{\left(\text{a}1,\text{t}\right)}=\left(1-\text{w}\right){\text{Q}}^{\text{M}\text{F}}{}_{\left(\text{a}1,\text{t}\right)}+\text{w}{\text{Q}}^{\text{M}\text{B}}{}_{\left(\text{a}1,\text{t}\right)}+\text{p}\cdot \text{S}\text{t}\text{a}{\text{y}}_{\left(\text{a}1,\text{t}\right)}$$ (4)

Q^MF^ refers to the first-stage bandit Q-value calculated in the SARSA algorithm. Stay_(a1,t)_*ϵ*{0,1} denoted whether a_1_ was taken in the previous trial multiplied by *p* (free parameter) estimating the tendency to repeat the previous action regardless of reward (i.e., perseveration). The probability of a first/second action was determined using a softmax, with a 1/β parameter representing the decision temperature:

$$P\left(a1,t\right)=\frac{\exp\left(\beta Q^{net}\left(a1,t\right)\right)}{\sum_{a'}\exp(\beta Q^{net}\left(a^{\prime },t\right))}$$ (5)

$$P\left(a2,t\right)=\frac{\exp\left(\beta Q^{MF}\left(a2,t\right)\right)}{\sum_{a'}\exp\left(\beta Q^{MF}\left(a',t\right)\right)}$$ (6)

The overall model included five parameters: a w-parameter (model-based/model-free trade-off), a learning-rate (α, the updating rate of values given a new outcome), a decision temperature (β, the amount of decision stochasticity), an eligibility trace (λ, referring to updating first-stage decision values based on second-stage outcomes) and a choice-perseveration parameter (*p*, tendency to repeat first-stage choices regardless of previous outcome or transition).

#### w-parameter II (DDM-RL, choice & RT)

While many reinforcement learning models are designed to explain agents’ choices, the value differences between two choices also influences decision-time [33,34]. Moreover, it has been argued that use of RTs can increase the reliability of parameter estimates in RL models [27]. To explore whether including RTs improve reliability of w-parameter estimates, we extended the Daw et al., 2011 RL model to predict a combination of choice and RT, based on an assumption that value discriminability will be reflected in both choice and RT (with higher value differences leading to quicker RTs).

Previous studies have argued that a combination of choice and RT can be predicted by integrating Q-learning RL algorithms with evidence accumulation mechanisms [27,34–37]. Here, we used the Wiener drift-diffusion model whereby the decision process is described as a continuous random walk (or diffusion) process [38–40]. In this model, evidence accumulated towards one of two boundaries, with time to reach one of the two boundaries and the identity of the attained boundary, determining decision-time and choice. At each time point, the amount of evidence in favour of one of the two alternatives drifted as a function of:

$$\frac{d}{di}X_{i}\;\sim \;Normal\left(\delta ,s^{2}\right)$$ (7)

where δ was a drift-rate towards the selected action, and *s*^2^ is fixed to 1 to allow scaling. The distance between the boundaries was determined by a free parameter–*a*, which represented the response policy. Greater distance between boundaries (*a* parameter) led to a more cautious policy (slower and more accurate response) while faster drift-rate (δ parameter) led to greater sensitivity (quicker and more accurate decisions). Finally, X_0_ (the amount of evidence when the process starts) was equal to 0.5*a, given that we assumed no prior bias towards one of the two alternatives. The time of first passage, and which boundary was attained first, were therefore probabilistic with a probability determined by the parameter set.

Next, to account for a combination of choice and decision-time generated by the value differences, Eq 7 was adjusted so that the drift-rate for each trial was the difference between the Q-values of the two alternatives, and the upper/lower boundary represented the selected and competitor choices a and a’ [27,34]:

$$\frac{d}{di}X_{i}\;\sim \;Normal\left(b\left(Q\left(a,t\right)-Q\left(a',t\right)\right),s^{2}\right)$$ (8)

Therefore $,\;\delta \;=\;b(Q\left(a,t\right)-Q\left(a^{'},t\right))$, meant a greater value difference between the selected and alternative action translates in the model as higher drift-rates. The *b* parameter allowed further scaling (as both fixing *s*^2^ = 1 and the Q-value range is arbitrary). Finally, a non-decision time parameter τ was added to the decision-time representing the duration that passed without any diffusion process (e.g., early perceptual/late motor processes), with RT being the sum of decision-time (accounted for by the first passage of time in the Wiener diffusion model) and τ. Overall, the current drift-diffusion model with reinforcement learning (DDM-RL) included five parameters from the Daw model accounting for RL algorithms (α_1_, α_2_, λ, *p*,w) along with three DDM parameters (*b*,*a*,*τ*). Estimation of log-likelihood given a combination of choice and RT per trial was obtained by using an analytic solution for Wiener’s first passage of time [38] (the code was obtained from [41]).

### Model-agnostic estimates for model-based/free trade-off

While the w-parameter serves as a process-based measurement of model-based/free trade-off (and thus has a clear interpretation), the literature makes use of model-agnostic measures that map directly to changes in the w-parameter. Model-agnostic measures can be beneficial as they are straightforward to calculate (can be done without model fitting or RL algorithms). Here we examined a commonly used score based on first-stage choice [4,18,23,30], describing a greater tendency of a model-based agent to revisit a state that was previously rewarded as a function of common vs. uncommon transitions. We show that this tendency translates into systematic value differences in the second-stage. Giving the known relationship between discriminability and RT [33,42] this observation allowed us to elaborate a second score based on second-stage RTs (RT_2_) [4,30].

#### MB-I (choice)

Model-based choice is estimated by calculating the interaction effects of transition (common vs. rare) and reward (rewarded vs. non-rewarded) in the previous trial on the probability of repeat of a first-stage choice in the next trial. A purely model-free learner is blind to transition structure, and therefore should display a higher probability of repeating the last first-stage choice when rewarded, regardless of transition (reward’s main effect, see S1 Fig, left panel). In contrast, a model-based learner has knowledge of transition probabilities, and makes use of that knowledge to choose the best bandit on the next trial. For a model-based learner a reward increases the probability of repeating a first-stage choice only when the previous transition was common, while after uncommon transitions reward reduces the probability of repeating first-stage action (increasing the chance that the agent would return to the state where the reward was obtained). This translates into a positive interaction of previous reward and previous transition on the probability of repeat of the first-stage action (see S1 Fig, right panel).

Let transition and reward at trial *t* be represented by X_transition*(t)*_*ϵ*{0,1} for common and uncommon and X_reward*(t)*_*ϵ*{0,1} for unrewarded and rewarded trials. Sticking with the same choice at the first-stage can be represented by Y_stay_ *ϵ*{0,1} for switch and stay choices. Then stay probability (chance of repeating first-stage choice) can be defined as P(Y_stay_ | X_transition(t),_ X_reward(t)_). Individual MB-I_(choice)_ scores can then be obtained by calculating the paired interaction of previous transition and reward on stay probability. This was achieved by first calculating the effect of transition separately for rewarded and unrewarded trials:

$$\text{R}\text{e}\text{w}\text{a}\text{r}\text{d}\text{e}\text{d}\;\;\;\;=\;\text{P}({\text{Y}}_{\text{s}\text{t}\text{a}\text{y}}{}_{\left(\text{t}+1\right)}|{\text{X}}_{\text{t}\text{r}\text{a}\text{n}\text{s}\text{i}\text{t}\text{i}\text{o}\text{n}}{}_{\left(\text{t}\right)}=0,{\text{X}}_{\text{r}\text{e}\text{w}\text{a}\text{r}\text{d}}{}_{\left(\text{t}\right)}=1)\;-\;\text{P}({\text{Y}}_{\text{s}\text{t}\text{a}\text{y}}{}_{\left(\text{t}+1\right)}|{\text{X}}_{\text{t}\text{r}\text{a}\text{n}\text{s}\text{i}\text{t}\text{i}\text{o}\text{n}}{}_{\left(\text{t}\right)}=1,{\text{X}}_{\text{r}\text{e}\text{w}\text{a}\text{r}\text{d}}{}_{\left(\text{t}\right)}=1)$$ (9)

$$\text{U}\text{n}\text{r}\text{e}\text{w}\text{a}\text{r}\text{d}\text{e}\text{d}\;=\;\text{P}({\text{Y}}_{\text{s}\text{t}\text{a}\text{y}}{}_{\left(\text{t}+1\right)}|{\text{X}}_{\text{t}\text{r}\text{a}\text{n}\text{s}\text{i}\text{t}\text{i}\text{o}\text{n}}{}_{\left(\text{t}\right)}=0,{\text{X}}_{\text{r}\text{e}\text{w}\text{a}\text{r}\text{d}}{}_{\left(\text{t}\right)}=0)\;-\;\;\text{P}({\text{Y}}_{\text{s}\text{t}\text{a}\text{y}}{}_{\left(\text{t}+1\right)}|{\text{X}}_{\text{t}\text{r}\text{a}\text{n}\text{s}\text{i}\text{t}\text{i}\text{o}\text{n}}{}_{\left(\text{t}\right)}=1,{\text{X}}_{\text{r}\text{e}\text{w}\text{a}\text{r}\text{d}}{}_{\left(\text{t}\right)}=0)$$ (10)

then calculating the difference between the two effects:

$$\text{M}\text{B}-{\text{I}}_{\left(\text{c}\text{h}\text{o}\text{i}\text{c}\text{e}\right)}=\;\text{R}\text{e}\text{w}\text{a}\text{r}\text{d}\text{e}\text{d}\;-\;\text{U}\text{n}\text{r}\text{e}\text{w}\text{a}\text{r}\text{d}\text{e}\text{d}$$ (11)

Scores that are constrained by group prior (i.e., hierarchical MB-I_(choice)_) were obtained by fitting a mixed-effect logistic for trial-by-trial data (the following indicates Wilkinson notation [43]):

$$\text{P}({\text{Y}}_{\text{s}\text{t}\text{a}\text{y}}{}_{\left(\text{t}+1\right)})\sim \;\text{T}\text{r}\text{a}\text{n}\text{s}\text{i}\text{t}\text{i}\text{o}{\text{n}}_{\left(\text{t}\right)}*\;\text{R}\text{e}\text{w}\text{a}\text{r}{\text{d}}_{\left(\text{t}\right)}+\;\left(\text{T}\text{r}\text{a}\text{n}\text{s}\text{i}\text{t}\text{i}\text{o}{\text{n}}_{\left(\text{t}\right)}*\;\text{R}\text{e}\text{w}\text{a}\text{r}{\text{d}}_{\left(\text{t}\right)}|\;\text{S}\text{u}\text{b}\text{j}\text{e}\text{c}\text{t}\right)$$ (12)

Whereby the MB-I_(choice)_ score was the individual slope of the transition x reward interaction_._

#### MB-II (RT)

Here, we added a less traditional MB score to ascertain whether this score increased the reliability of MB estimates [4,30]. This score is based on an assumption that more model-based participants are quicker to make a second-stage choice after common transitions, and slower after uncommon transitions, compared to model-free participants. This score was previously found to correlate positively with MB-I_(choice)_, and w-parameter [4,30], as well as correlate with right ventral striatum dopamine levels during task performance, mimicking an effect obtained with model-based model parameters [30]. The score was obtained as follows:

$$\text{M}\text{B}-\text{I}{\text{I}}_{\left(\text{R}\text{T}\right)}=\text{m}\text{e}\text{a}\text{n}\left(\text{R}{\text{T}}_{2}|\;{\text{X}}_{\text{t}\text{r}\text{a}\text{n}\text{s}\text{i}\text{t}\text{i}\text{o}\text{n}(\text{t})}=1\right)\;-\;\text{m}\text{e}\text{a}\text{n}\left(\text{R}{\text{T}}_{2}|\;{\text{X}}_{\text{t}\text{r}\text{a}\text{n}\text{s}\text{i}\text{t}\text{i}\text{o}\text{n}(\text{t})}=0\right).$$ (13)

with MB-II_(RT)_ scores that are also constrained by group parameter obtained by a mixed effect linear regression whereby MB-II_(RT)_ was the individual slope of the transition main effect (the following indicates Wilkinson notation [43]):

$$\text{R}{\text{T}}_{2(\text{t})}\sim \;\text{T}\text{r}\text{a}\text{n}\text{s}\text{i}\text{t}\text{i}\text{o}{\text{n}}_{\left(\text{t}\right)}{+}_{}\left(\text{T}\text{r}\text{a}\text{n}\text{s}\text{i}\text{t}\text{i}\text{o}{\text{n}}_{\left(\text{t}\right)}|\;\text{S}\text{u}\text{b}\text{j}\text{e}\text{c}\text{t}\right)$$ (14)

In the results section below, we test the relationship between process-based and model-agnostic estimates which further allowed us to describe why stronger deployment of model-based strategies in the first-stage might lead to higher MB-II_(RT)._

## Results

### Obtaining model-based estimates from empirical data

#### Psychometric properties in empirical data

We used data from the Neuroscience in Psychiatry Network’s (NSPN) study [28]. This is a community-based longitudinal sample of young volunteers (age 14–24 years), living in Cambridgeshire and London regions, UK. The study was designed to measure developmental change. Participants completed a two-step task lab testing, among other measurements. Here we focused on psychometric properties of the task, across questions referring to development. Participants were excluded if they had recognised learning disabilities or neurological disorders. Our final dataset included 554 subjects (274 males, 280 females) at two time points: baseline (mean age = 18.85, range 14.1 to 24.98) and follow-up (mean age = 20.33, range 15.11 to 26.48). For further details see Methods as well as Kiddle et al., 2018.

#### Process-based estimates

To obtain w-parameters estimates at the individual level, we fitted the RL (choice) and DDM-RL (choice & RT) to empirical data, separately for each time point. Fitting was performed either with (i.e., hierarchical fit) or without group priors (i.e., individual fit, see Methods for further details). We fit two versions of the RL model, with five parameters (single α and β for both stages) and seven parameters (α_1_, α_2_, β_1_, β_2_ for each stage), and compared the two by calculating Bayesian inference criteria (BIC_int_) which penalizes for the number of parameters (difference of 10 points or more is considered strong evidence, with lower scores indicating better fit)[44]. BIC at both baseline [BIC _int_5_parameters_ = 134738.6, BIC _int_7_parameters_ = 134194.7] and follow-up [BIC _int_5_parameters_ = 216901.9, BIC _int _7_parameters_ = 215646] favoured the seven parameter model. Hence, this model was used for all further analysis (see S2 Table for parameters descriptive statistics).

We also fit two versions for the DDM-RL, one with single DDM parameters (a,b,τ) at each stage and one with two sets (a_1_,b_1_,τ_1_,a_2_,b_2_,τ_2_) at each stage. BIC at both baseline [BIC _int_8_parameters_ = 113648.1, BIC _int _11_parameters_ = 104220.4] and follow-up [BIC _int_8_parameters_ = 174546, BIC _int_11_parameters_ = 162964.3] favoured the 11 parameter model. Hence, this model was used on further analysis (see S2 Table for parameters descriptive statistics).

#### Model-agnostic estimates

We next calculated model-agnostic scores, MB-I_(choice)_ and MB-II_(RT)_ for each individual from choice and RT data. We calculated the scores separately at each time point either with (i.e., hierarchical scores, Eqs 12 and 14) or without group priors (i.e., individual scores, see Eqs 11 and 13 and S2 Fig for histograms). To obtain an estimate of the effect-size of model-agnostic measures we calculated η^2^ estimates (explained variance) for each score, across participants and across time measurements. For MB-I_(choice)_ the transition*reward interaction factor explained 25.1% of the first-stage stay probability. For MB-II_(RT)_, the transition factor explained 67.3% of the mean RT_2_ variability.

### The relationship between process-based and model-agnostic estimates

**Fig 2:**
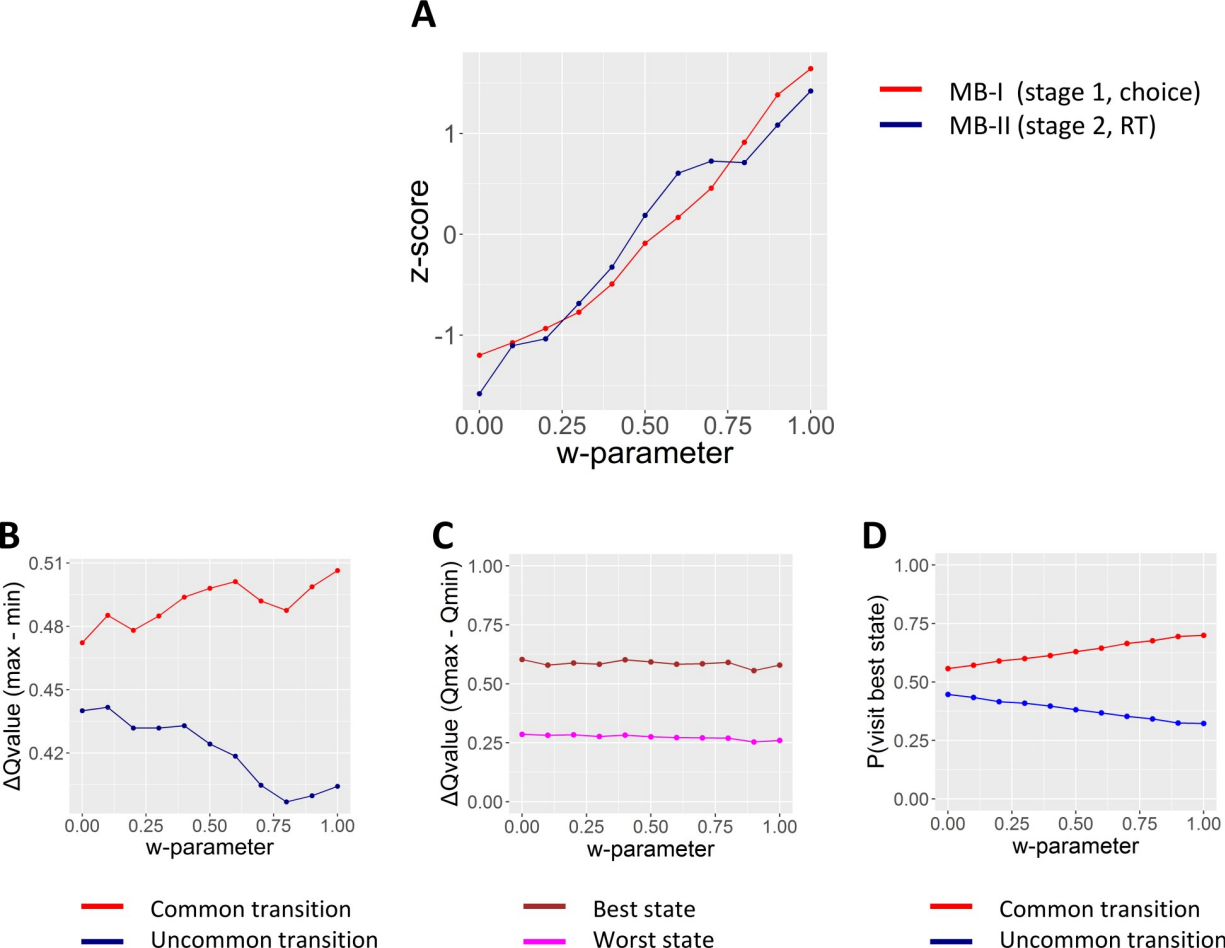
Examining the relationship between model-agnostic scores (MB-I_(choice)_, MB-II_(RT)_) and the w-parameter. To obtain these plots we gradually increased the w-parameter from 0 to 1 in .1 steps, each time simulating 200 experiments with 5,000 trials each using the DDM-RL model (All other parameters were selected randomly and uniformly form a pre-defined range, of α_1/2_[0,1], λ[0,1], w[0,1], p[0,.5], b_1/2_[1,10], a_1/2_[1,3], τ_1/2_[.01,.5] for each experiment). (**A**) For each of the 200 experiments, we averaged MB-I_(choice)_ and MB-II_(RT)_ (see Eqs 11 and 13) scores. We then standardized the eleven mean scores, separately for each MB score. Results showed a strong relationship between the w-parameter and both model-agnostic measures. (**B**) Here we illustrate how deployment of model-based strategies in the first-stage is affecting MB-II_(RT)_ via systematic effects on second-stage value discrimination. Specifically, Panel B presents averaged ΔQ-value (max–min Q-value) for the second-stage state the agent visited. Results confirmed that higher w-parameter values lead to higher/lower value discriminability (ΔQ-value) after common/uncommon transitions, respectively. Notably, in the DDM-RL model ΔQ-values are directly and positively associated with drift-rates and hence contribute to faster RTs (see Eq 8). This result illustrate why higher w-parameter is associated with quicker/slower RT_2_ after common/uncommon transitions, respectively. (**C/D**) To further demonstrate how deployment of model-based strategies in the first-stage leads to systematic value differences in the second-stage we labelled in each trial the best and worst state (state that included the highest Q-value out of the four available second-stage bandits, and the alternative state). Panel C shows that across all simulation the best state was related with higher value discriminability (higher ΔQ-value), regardless of the w-parameter. Panel D further shows that higher w-parameter is related with higher probability of visiting the best state by means of common transitions (see Eq 3). Therefore, Panels C & D illustrates the reason that higher w-parameter leads to higher value discriminability after common trials as illustrated in Panel B.

First, we wanted to assess whether changes in w-parameter directly affected MB-I_(choice)_, and MB-II_(RT)_. To do this we systematically increased the w-parameter in an RL-DDM model, and calculated both MB-I_(choice)_ and MB-II_(RT)_, in-silico. A strong relationship between all three estimates was evident (see Fig 2A).

While the relationship between the w-parameter and MB-I_(choice)_ is well documented [18], that is not the case for MB-II_(RT)._ This raises a question as to why does RT_2_ differ as a function of model-based/free trade-off? Our assumption was that compared to a model-free agent, a model-based agent has better discriminability between the values of the two options (larger Q-value differences) when reached by a common vs. an uncommon transition. This is because a model-based agent is more likely to choose a first-stage bandit that will lead to the maximal Q-value out of the four second-stage alternatives via a common transition (Eq 3 and Fig 2D). Therefore, common transitions for model-based agents have a better chance of having a larger Q-value difference and hence better discriminability between the two values (see Fig 2B), whereby higher discriminability is tightly related to quicker RTs. To test this assumption we performed two complimentary in-silico analyses. First, we located in each trial the second-stage state that had the highest Q-value bandit out of the four second-stage bandits (hereafter, ‘best state’) and the alternative state (hereafter, ‘worst state’). We then calculated the averaged ΔQ-value (maximum–minimum Q-value) for the best vs. worst state and compared the two ΔQ-values for the two states. We found that on average the best state also had better value discriminability (mean ΔQ-value = .59) compared to the worst state (mean ΔQ-value = .27; see Fig 2C). This confirms the first part of our assumption, postulating that the state that holds the highest Q-value bandit (out of the four bandits) also entails higher value discriminability. Second, since model-based agents are more likely to reach the state with the highest Q-value bandit by means of a common transition (see Eq 3 and Fig 2D), it follows that higher w-parameter will lead to higher ΔQ-value for common vs. uncommon transitions. A second analysis confirmed this assumption, showing higher ΔQ-value for common and lower ΔQ-value for uncommon transitions as w-parameters values were increased in-silico (see Fig 2B). In the RL-DDM model ΔQ-value is directly translated into higher drift-rates, leading to quicker RTs on average (see Eq 8). Thus, this result confirms our assumption that systematic differences in model-predictions for RT_2_ as a function of transition type, are a direct result of model-based/free trade-off at the first-stage.

**Fig 3:**
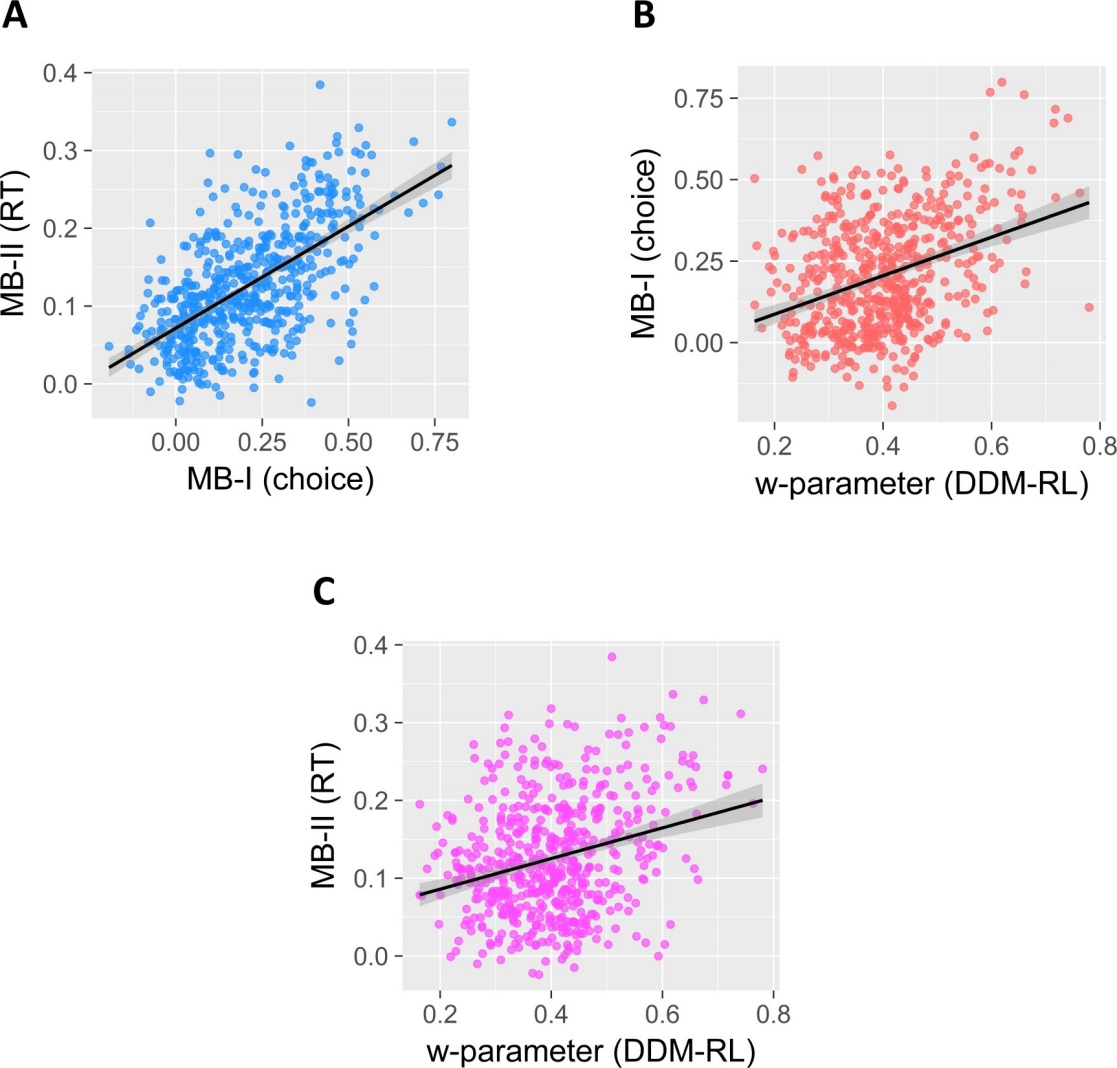
(**A/B/C**) Scatterplots showing the relationship between the three hierarchical model-based estimates obtained from empirical data (scores were averaged across baseline and follow-up).

To examine the relationship between the three scores in empirical data, MB-I_(choice)_, MB-II _(RT)_ and w-parameter we averaged each score across both time points (baseline and follow-up) and calculated Pearson/Spearman correlation coefficient (see Table 1). Results suggest a strong relationship between MB-I_(choice)_, MB-II _(RT)_ (with ~37% shared variance, see Fig 3A), and a moderate relationship of both with w-parameter (see Fig 3B and 3C). Examining Table 1 shows that hierarchical scores outperform individual ones in general. Finally, we examined whether correlations between the w-parameter and the scaling (*b*_2_), or the threshold (a_2_) parameters in the second-stage contribute to the empirical correlations between the w-parameter and MB-II_(RT)_. For example, if model-based individuals also had systematically larger thresholds and/or systematically higher scaling in the second-stage this might inflate MB-II_(RT)_. We calculated Spearman correlations and found a modest positive correlation between w and b_2_ (r = .09, p = .03) and a non-significant correlation between a_2_ (r = .06, p = .14). Importantly, Spearman partial-correlation analysis indicated that the empirical relationship reported in Table 1 between the w-parameter and MB-II_(RT)_ remains largely unaffected after controlling for b_2_ parameter estimates (Partial correlation = .41, p < .001).

**Table 1:** Correlation estimates describing the relationship between the different model-based estimates.

	MB-I (choice)	MB-II (RT)
**Individual scores:**		
MB-II (RT)	.53^a^_(.47-.59)_	.
w-parameter (RL)	.38 _(.30-.45)_	.26 _(.18-.34)_
w-parameter (DDM-RL)	.41 _(.33-.47)_	.24 _(.16-.32)_
**Hierarchical scores:**		
MB-II (RT)	.61^a^_(.56-.66)_	.
w-parameter (RL)	.31^b^_(.23-.38)_	.33^b^_(.26-.41)_
w-parameter (DDM-RL)	.37^b^_(.30–44)_	.36^b^_(.29-.43)_

Note.

^a^Pearson correlation estimate.

^b^Spearman rank estimate

### Parameter recovery

**Fig 4:**
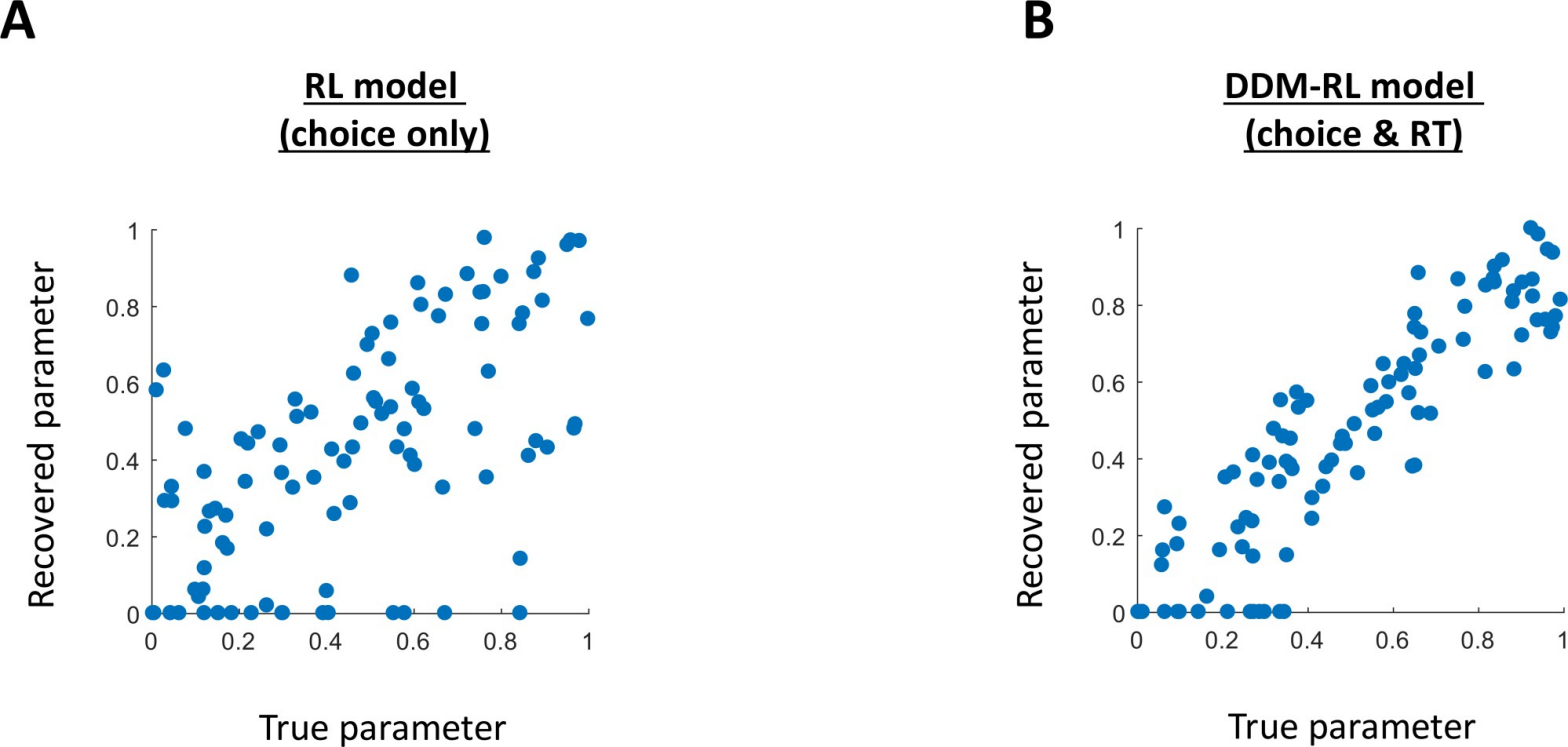
(**A/B**) Scatter plots for true compared to recovered w-parameter (estimating model-based/free trade off). Results show a better correlation for DDM-RL (panel B; modeling choice & RT, r = .9) compared with an RL (choice only) model previously reported in the literature (panel A, r = .62).

To examine the influence of task length on the reliability of estimated parameters, we performed a parameter recovery analysis. Table 2 presents the correlation between the true and recovered parameters, as a function of the number of trials in the analysis. While the w-parameter reached an acceptable value only after ~1000 trials for the RL model, the DDM-RL model reached the same value after as little as 200 trials (see Table 2 and Fig 4A and 4B). Furthermore, the fact that the learning rate for the first-stage showed better recovery for the DDM-RL vs. RL model, suggests an overall better parameter recovery for the first-stage in the former.

**Table 2:** Spearman's correlation estimating the relationship between the true and recovered parameters.

		Trials in the analysis			
		200	500	1000	5000
RL model (choice)	α_1_	.54	.62	.68	.92
	α_2_	.95	.98	.99	.99
	λ	.53	.71	.71	.88
	***w***	**.61**	**.69**	**.82**	**.97**
	*p*	.82	.90	.91	.97
	β_1_	.82	.90	.93	.98
	β_2_	.89	.96	.98	.99
DDM-RL model (choice & RT)	α_1_	.68	.72	.84	.94
	α_2_	.99	.99	.99	.99
	λ	.58	.75	.83	.92
	***w***	**.90**	**.95**	**.96**	**.99**
	*p*	.91	.94	.97	.99
	*b*_1_	.93	.93	.99	.99
	*a*_1_	.93	.98	.99	.99
	τ_1_	.99	.99	.99	.99
	*b*_2_	.99	.99	.99	.99
	*a*_2_	.97	.99	.99	.99
	τ_2_	.99	.99	.99	.99

### Internal stability

To measure internal stability, we calculated split-half reliability scores [45–47]. These scores are obtained by splitting a task into two (or more) parts and then estimating the extent to which the different parts reflect the same score. Here, we adopted a common practice of splitting the task into odd and even trials [45,46]. Note that we can still calculate MB score even when omitting odd/even trials by omitting the behaviour of the previous trial from the analysis, but not the coding (rare/common, rewarded/unrewarded). We estimated a MB score separately for each part, calculated a Pearson correlation coefficient to estimate the extent that the two parts reflect the same score, and used the Spearman-Brown formula to get a final estimate of internal constancy (owing to splitting the task into two parts, the Pearson correlation scores reflects internal consistency for only half of the trials; Spearman-Brown formula allows correction of the correlation estimate to reflect internal reliability as if it was obtained in two parts, each with a complete number of trials). A conventional criterion is that the odd-even correlation should exceed .7. Table 3 summarises our findings showing good reliability for MB-I_(choice)_, when using hierarchical scores, and for MB_(RT)_ for both individual and hierarchical scores.

**Table 3:** Psychometric properties for model-based estimates.

		Internal consistency (201 trials)	Temporal stability
MB-I _(choice)_	Individual scores	.52^c^_(.45–58)_	.28^a^_(.20-.36)_
	Hierarchical scores	.81^c^_(.78-.84)_	.40^a^_(.32-.46)_
MB-II _(RT)_	Individual scores	.87^c^_(.85-.90)_	.33^a^_(.25-.40)_
	Hierarchical scores	.87^c^_(.85-.89)_	.33^a^_(.25-.40)_
Latent score (choice & RT)		.	.75^a^_(.71-.78)_
w-parameter (RL model)	Individual scores	.	.16 ^b^ ^(.07-.24)^
	Hierarchical scores	.	.21^b^_(.13-.29)_
w-parameter (DDM-RL model)	Individual scores	.	.20^b^_(.12-.28)_
	Hierarchical scores	.	.14^b^_(.05-.22)_

^a^Pearson correlation estimate.

^b^Spearman rank correlation estimate.

^c^Spearman-Brown corrected Pearson correlation estimate.

Estimates in brackets represent 95% confidence intervals.

**Fig 5:**
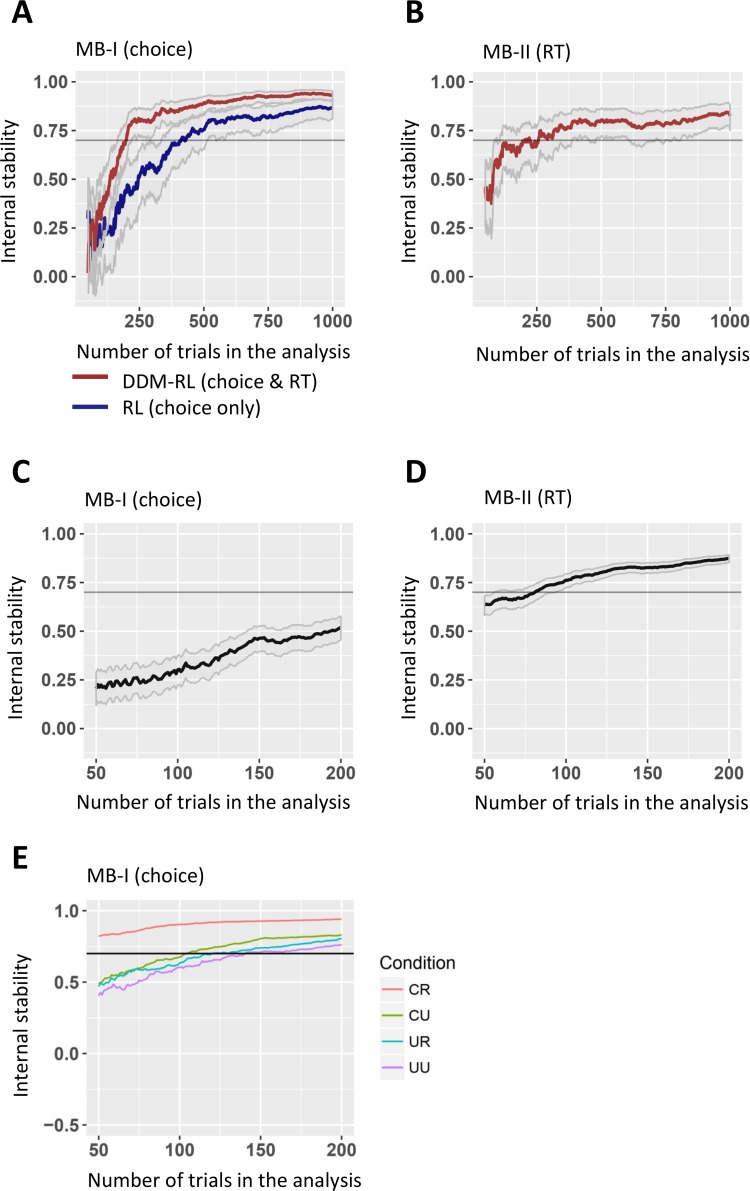
Internal consistency estimates for MB-I_(choice)_ and MB-II_(RT)_. In all figures, x-axis represents the number of trials in the analysis, and y-axis the Pearson’s correlation (corrected using Spearman-Brown formula) between the scores calculated for odd and even trials. (**A**) Internal stability for MB-I_(choice)_ obtained from simulated data of RL vs. DDM-RL models. Results suggest that reliability reached criteria for the RL-DDM with fewer trials compared to the RL model. (**B**) Internal stability for MB-II_(RT)_ obtained from simulated data of the DDM-RL model. (**C/D**) Internal stability for MB-I_(choice)_ and MB-II_(RT)_ calculated from empirical data (follow-up only). (**E**) Internal consistency in empirical data for the four conditions that assemble MB-I_(choice)_ (CR: common-rewarded, CU: common-unrewarded, UR: uncommon-rewarded, UU: uncommon-unrewarded, see Eq 9–11). Ribbons present 95% CI. The horizontal line represents the .7 criteria for internal stability.

To test for the effect of the number of trials on internal stability, we performed the half-test reliability analysis using the first 20 trials alone. We then repeated the analysis, each time adding one additional trial and re-calculating the reliability scores. Fig 5 presents our findings for empirical data (only follow-up data, where we had more trials), as well as simulated data from the DDM-RL and RL models. We found that MB-I_(choice)_ score first reached criteria (>.7) after 411 trials for data simulated from the RL model. MB-I & II reached the same criteria after 199 and 204 trials, respectively, for data simulated from the DDM-RL model. In empirical data, MB-I_(choice)_ failed to reach stability after 200 trials. If indeed an evident linear trend is maintained then the reliability of the MB-I_(choice)_ should reach ~.8 after roughly 350 trials. Fig 5 suggests that the same internal consistency (.8) for MB score II (RTs) can be reached after about 100 trials.

One reason MB-I_(choice)_ might have lower internal stability is that it reports on four types of trials (see Eqs 9–11). Moreover, uncommon trials are less frequent by definition, and so these estimates are potentially noisier. Consequently, we examined the internal consistency of stay probability for these four trial types separately, again using the follow-up data alone (where we had more trials).

We found for all four types an acceptable internal stability after 200 trials (see Fig 5). Why would MB-I_(choice)_ score show low internal consistency after 200 trials, if the four conditions used to calculate it are reasonably stable? The answer may rest in the observation that difference scores are mathematically less reliable than their components, as long as the measurement noise is independent [48]. This means that only high reliability for each of the four conditions will provide reasonable reliability for the MB score itself. Therefore, it is plausible that after ~350 trials, when the four probabilities attain good internal reliability, will an interaction score become acceptably reliable.

### Temporal stability

To assess temporal stability we calculated Pearson’s correlation between baseline and follow-up MB estimates. Results are presented in Table 3. We found overall low temporal stability for the w-parameter in both RL and DDM-RL models (see S3 Table for the remaining parameters). Model-agnostic scores had low to medium stability with slightly better estimates for hierarchical MB-I_(choice)_ scores compared with individual ones. We also applied a method suggested by Silva and Hare, allowing to correct for a minority of cases where the state value can cause misinterpretation of MB-I_(choice)_ [49]. Specifically, we added two control variables to the hierarchical regression coding the fractals selected at the first-stage and second-stages (See Eq 2 in Silva and Hare study [49]), however we found very similar temporal stability (r = .41) for MB-I_(choice)_.

To assess whether better temporal stability were obtained by aggregating the two MB scores (choice and RTs) we turned to latent factors analysis using structural equation modelling (SEM) [50]. SEM is a multivariate method that combines factor analysis and multiple regression. It allows estimation of structural relationships between latent constructs and their measured variables (indicators). Latent factors are considered less noisy than their counterparts [51], but come with the disadvantage that these latent factors are sometimes difficult to interpret [52]. Here, we constructed two MB latent factors for baseline and follow-up time points, with each latent factor predicting MB-I_(choice)_ and MB-II_(RT)_. Pearson correlation between the baseline and follow-up latent MB factors, showed better estimates compared to the separate scores (Table 3, see also S3 Fig for indicators).

Finally, as the time-gap between measurements was not the same for all participants (see Procedure), we explored whether temporal reliability might increase with shorter time-gaps. We calculated a regression where baseline scores, time-gap and their paired interaction predicted follow-up scores (separately for MB I & II). For the individual MB-I_(choice)_ we found a significant interaction (p < .01), such that a shorter time-gap predicted higher temporal stability. To assess further the magnitude of this interaction effect, we performed a median split on time-gap estimates (above/below 18 months between baseline and follow-up). We found that participants in the short time-gap group had higher temporal stability, (r = .41) compared with those tested on longer time-gaps (r = .13). No interaction effect of time-gap on temporal stability estimates was found for hierarchical regression MB-I_(choice)_ or MB-II_(RT)_ scores (all p-values>.8). Finally, our data set had a subset of 61 participants that had an additional measurement, six months after baseline. S1 Text presents temporal stability scores between baseline and the six-month follow-up.

### Power analysis for group level estimates

A question of great practical interest is how reliability in group-level estimates scale with group size. Specifically, an interest in group differences in model-based abilities (e.g. assessing differences between clinical and healthy populations, or effects of manipulations) renders it important to estimate the chance of finding a between-subjects effect in group comparisons, given such an effect exists in the population. We performed a power analysis using simulated data from the DDM-RL model, examining an ability to detect differences in MB estimates between two groups (control vs. experiment). Specifically, we examined statistical power (chance for a statistically significant result given an effect exists in the population) as a function of effect-size between groups (small, medium or large), the number of participants (30, 100 or 500 per group) and the number of trials (200, 500 or 1000).We generated two truncated w-parameter Gaussian distributions (control, experiment) with a standard deviation of .1 (based on the empirical parameters obtained from fitting the model, see S2 Table) and means set to generate a small effect-size (group means were .49/.51, Cohen’s d = .2), medium effect-size (group mean was .475/.525, Cohen’s d = .5) or large effect-size (group means .46/.54, Cohen’s d = .8). For each of the three effect-sizes we sampled across 1000 iterations of N participants (30, 100 or 500) with n trials (50, 100 or 500).

For each iteration we calculated MB I&II scores (choice & RT) for each agent, and calculated the test statistics chi-square of a MANOVA analysis with both scores as dependent variables. A statistically significant chi-square means that an H0 (no group difference in model-based estimates) can be rejected given the data. As we know the ground truth (H0 is false), we calculated the percentage of studies that would reach the correct conclusion (reject H0). Therefore, for each effect-size, sample size (N) and experiment length (n) we calculated the percentage of samples above the critical chi-square, representing the proportion of studies that would obtain a statistically significant effect. Table 4 presents the results, showing very low power for small effect sizes for all sample sizes, reasonable power for a medium effect with 500 subjects, and good power for a large effect with 500 subjects.

**Table 4:** Statistical power (percent of studies that rejected the null hypothesis, given an effect exists) for a between group design (control vs. experiment). Table values show the chance of finding a statistically significant between group effect as a function of true effect-size, sample-size and number of trials in the experiment.

		200 trials	500 trials	1000 trials
**Small effect** **(Cohen’s d = .2)**	30 participants	4.5%	5.8%	7.5%
	100 participants	6.2%	9%	8.2%
	500 participants	14.1%	24%	28.2%
**Medium effect** **(Cohen’s d = .5)**	30 participants	7%	7%	7.9%
	100 participants	11%	15%	15.1%
	500 participants	52.8%	64%	67%
**Large effect** **(Cohen’s d = .8)**	30 participants	11.5%	10.9%	13%
	100 participants	31.5%	30.3%	37.8%
	500 participants	94.7%	90.7%	96.9%

## Discussion

Our study examined the psychometric properties of model-based/model-free estimates in a two-stage decision task. First, we quantified the extent to which participants consider both the transition and reward history when making a first-stage choice (MB-I_(choice)_). Second, we quantified the benefit/cost in second-choice RTs following common/uncommon transitions (MB-II_(RT)_).

While MB-I_(choice)_ is widely exploited in the literature [4,13,18,30], the latter is less used [4,30], raising the question as to whether it actually helps quantify MB processes and whether it can enhance the reliability of MB estimates. We considered a computational model that predicted both choice and RT (DDM-RL). A simulation analysis showed a close relationship between the process-based w-parameter (quantifying model-based/free trade-off at the first stage) and MB-II_(RT)_. Specifically, we found that deployment of model-based strategies at the first-stage affected the value discriminability of bandits at the second-stage. As discriminability is strongly and negatively related to RTs [33,42], it follows that these value differences should be observed at the level of RTs. A tight relationship between the w-parameter and MB-II_(RT)_ highlights the latter as a promising index of the deployment of model-based strategies at the first-stage. Furthermore, our findings suggest that a model that predicted a combination of choice and RT had better recovery properties for the w-parameter, as well as greater internal consistency for model-agnostic measures in simulated data (both choice and RT scores).

What do our results suggest in terms of reliability of model-agnostic MB measures? First, we found that MB-I_(choice)_, but not MB-II_(RT)_ had a low internal stability when the 201 trials version of the task was used. An antecedence search for Daw et al.’s (2011) study revealed 31 studies that directly used the same paradigm to quantify model-free and model-based involvement from observed data. Of these 31 studies, 25 studies used 201 trials or less, and the remainder used approximately 300 trials. The low internal reliability of MB-I (choice) seems to be attributable to the fact that this score is based on estimation of four conditions. Moreover, the fact that uncommon trials are less frequent, renders it even more challenging. According to our calculations, MB-I_(choice)_ is expected to attain reasonable internal reliability at ~350 trials, at least 75% longer than is usual practice up to now. Second, as regards temporal stability, both MB-I_(choice)_ and II_(RT)_ fell short in terms of expected conventional criteria. The use of the group prior (hierarchical fit) vastly improved the internal consistency score for MB-I_(choice)_, and its temporal stability to a lesser extent. However this should be interpreted with caution, since the group prior might drive a more stable, but less valid measure. Finally, using both MB-I_(choice)_ and II_(RT)_ to estimate a latent model-based factor, showed much better stability between the two time points. This is in line with previous claims that latent factor analysis using SEM can be a simple and straightforward method of reducing measurement noise [51].

Overall, a DDM-RL model improved psychometric properties for model-based estimates in the two-step task as reflected in better parameter recovery, as well as improved stability of model-agnostic scores (calculated from simulated data). It also provided a link between model-based/free trade-off in first-stage choices and RT differences in second-stage choices. This link is important because it supports a claim that both choice and RT based model-agnostic scores reflect model-based/free trade-off at the first-stage, allowing use of the combination of these two scores to provide a more reliable estimation. However, when looking at the psychometric properties of the w-parameter, it seemed DDM-RL did not improve the temporal stability compared to that of the RL (choice only) model. One reason for this might be that the number of trials was not enough to allow DDM-RL to attain a stable estimation of the w-parameter (121 trials in baseline and 201 at follow-up). Another possible reason might be that model-based/free trade-off in the first stage influence RTs in the second-stage via additional processes not accounted for by DDM-RL. For example, MB-II_(RT)_ score might also be related to the expectation participants have for the common transition-related state. That is, participants with stronger deployment of model-based strategies in the first-stage, also take more time to make a decision regarding a second-stage selection, shortening RT for common trials. However, in the uncommon transition, model-based individuals would need to inhibit their expectation and switch to an unexpected state, thus prolonging response latency [53]. A model-free participant would be less sensitive to such expectations and show similar RTs on both transitions. However, DDM-RL model was unable to capture these set-shifting effects, therefore such possibilities need to the object of further study.

Two straightforward procedures can promote increased reliability in model-based estimates using the two-step paradigm. The first is relatively easy to implement, and involves acquiring more trials than commonly used in current practice. The second is consideration be given to using both first-stage choice and second-stage RTs to attain higher stability. Until now, most if not all decision models in the goal-directed literature use choice data, disregarding the time an agent takes to commit to that choice. Decision models that account for a combination of choice and RT (e.g., evidence accumulation models) might prove more reliable than models that rely on choice alone. Finally, where possible, the use of repeated measures indicating a latent model-based factor should be much preferred.

Given the noisy estimates for model-based behaviour, this raises questions concerning positive findings already reported (see introduction). Our findings would suggest that past studies underestimate the true effect of different contributions to choice behaviour. An example here is the small effect sizes found in studies that compare clinical and healthy participants [13,29]. While some studies report MB I & II [4,29,30], we suggest that use of a combination of these two scores in both model-agnostic and computational analyses should increase reliability of model-based estimates, allowing for better assessment of effect-sizes and boosting replicability.

In conclusion, a conceptual distinction between model-based vs. model-free processes in behaviour control has fostered a rich and growing literature. However, we highlight potential reliability issues that can be addressed by relatively simple measures, such as increasing the number of trials and by applying modelling to both choice and RTs. One goal of computational neuroscience is to assess cognitive processes at a single subject level. For example, predicting/explaining clinical symptoms based on computational/neurological cognitive estimates to eventually inform clinical decisions [54,55]. This requires provision of stable and reliable estimates and our study highlights ways this can be advanced.

### Limitations

Two limitations need to be considered. First, test-retest stability might suffer from the fact that, in our study, at the second measurement participants had more experience with the task. This can either reduce re-test stability (both measurements are not measuring exactly the same thing) and/or might increase the apparent reliability of the second measurement, as more experience in the task can lead participants to behave more consistently. Our analysis cannot differentiate between those aspects. Second, our data were obtained from an adolescent and young adult population and the study findings cannot be generalised outside the type of population we investigated.

## Materials and methods

### Ethics statement

The study was carried out in accordance with the Declaration of Helsinki and Good Clinical Practice guidelines. Ethical approval was granted by Cambridge Central Research Ethics Committee (12/EE/0250).

### Participants

Data was obtained from the Neuroscience in Psychiatry Network’s (NSPN) study [28]. This is a community-based longitudinal sample of young volunteers (age 14–24 years), living in Cambridgeshire and London regions, UK. The study was designed to measure developmental change. Participants were recruited by invitation sent to local general practitioners (GP), adverts in the community, schools and further education colleges. Written informed consent was given by the participants aged 16–24 years, those aged 14–15 years gave written informed assent and their parents/legal guardian provided written informed consent. Participants were recruited in an age- sex-stratified sample, for the following five age groups: 14–15, 16–17, 18–19, 20–21, and 22–24 years. Participants were invited to take part in detailed behavioural assessments including computer-based evaluations, clinical assessments and IQ measures, during three time points (N = 819, N = 63, N = 571). Only participants that had completed the measurement of interest (two-stage task) were included in the current analysis. In order not to reduce the sample-size substantially (owing to lower sample-size at the second time point) we used data only from the first and third time-points (henceforth, baseline and follow-up). However we report a subset of our analysis with the second time-point in S1 Text. Our final dataset included 554 subjects (274 males, 280 females) in two time points: baseline (mean age = 18.85, range 14.1 to 24.98) and follow-up (mean age = 20.33, range 15.11 to 26.48). Further details about recruitment, participants consent, and ethical approval can be found at Kiddle et al., (2018) [28] (Note that the Kiddle et al., study was reported before data collection was completed, and has a slightly lower amount of participants included. The current analysis is based on data collected up until March 2018. To the best of our knowledge, no more participants were tested after that date).

### Procedure

For both measurements (baseline and follow-up) participants were invited to a lab session in one of the UK’s collaborating institutions [28]. The mean time gap between the two measurements was 17.75 months (range 11.76 to 31.44 month). At each measurement session participants completed computer-based cognitive evaluations, clinical assessments and IQ measures. At the end of the assessment day, participants were paid a fixed amount plus a bonus based on performance. For the purpose of this study we focus on analysing data obtained from the two-stage task.

### Two-stage decision task

The task was the same as the one developed by Daw at el., (2011) [18], and is described in Fig 1. Participants are instructed to win as many rewards (play pounds) as possible, and were told also that they would receive a payment bonus based on overall task performance. At each of the stages, subjects select one of two stimuli within 2 seconds. The inter-trial interval was randomly selected from a uniformed distribution ranging from 1 to 2 seconds. The task included 121 trials at baseline and 201 trials at follow-up (a shorter version in baseline was given due to time constraints, and increased to 201 to match Daw et al., 2011). A short break was provided after half of the trials were completed.

### Participant exclusion and pre-processing

We included participants that had a completed data set for both baseline and follow-up (N = 569). We excluded participants that responded in the two-stage task with the same key on more than 95% of the trials (two participants), or had implausible RTs (below 150_ms_) on more than 10% of the trials (13 participants; Gillan et al., 2016). This resulted in the inclusion of a total of 554 participants in our full analyses. For the remaining two-stage task data, the first trial in each block, as well as trials with implausible RTs (below 150_ms_) were omitted from the analysis (1% of the overall trials).

### Parameter recovery

To perform parameter recovery analysis we randomly selected parameters values for 100 agents from uniform distribution with ranges set to α[0,1], β[1,8], λ[0,1], *w*[0,1], *p*[0,.5], b_1/2_[1,10], a_1/2_[1,3], τ_1/2_[.01,.5]. Parameters were extracted individually for each agent by optimizing a log-likelihood function with a genetic algorithm ‘GA’ optimization method in in Matlab, using a population size of 200 and a maximum iteration of 400.

### Model-fitting routines

Individual fit was obtained by using a genetic algorithm ‘GA’ optimization method with a population size of 200 and maximum iteration of 400, followed by *fminunc* optimisation using Matlab, separately for each individual data set. This was repeated five times for each individual, each round with different starting points. To obtain hierarchical fit we used expectation-maximisation with Laplace approximation method[56]. In this approach, individual-participant parameters are treated as independent random effects sampled from Gaussian-population distributions (one distribution per parameter), whose means and variances are estimated. To examine how well the model predicted participants’ RTs, we compared simulated and observed RT histograms, and found a good match (see S4 Fig, see also S5 and S6 Figs for model predictions and empirical behaviour as a function of value discriminability).

MB-I_(choice)_ and MB-II_(RT)_ scores were obtained separately for each time. Individual scores were the descriptive individual mean differences described in Eqs 11 and 13. That is, for MB-I_(choice)_ we coded a *Stay* variable (0 for switch, 1 for stay), and calculated the mean(*Stay*) for each of the four conditions in Eqs 9 and 10 (the previous trial was with common/uncommon transition, rewarded or unrewarded). MB-II_(RT)_ was the difference in mean RT_2_ for uncommon vs. common trials (Eq 13). We also obtained individual scores that were based on group priors (Hierarchical scores, Eqs 12 and 14). For MB-I_(choice)_ we calculated a logistic regression using ‘lmer’ R package, with a Laplace approximation and bound optimization by quadratic approximation (BOBQA). Variables coding was done similar to previous studies, with Transition coded as -1 or 1 for uncommon/common transitions, and Reward as -1/1 for unrewarded / rewarded trials[4,13,49]. The logistic regression included fixed effects and random effects for the full factorial design where *Stay* is predicted by intercept, previous transition, previous reward and their interaction. The random effects for the transition x reward interaction was then used as MB-I(choice). For MB-II_(RT)_ we fitted a mixed effect linear regression using the same R package, predicting RT_2._ The linear regression included fixed and random effect for the intercept and transition effects. Slope random effect for the transition effect was used for MB-II_(RT)_ scores.

### Structural equation modeling

Temporal stability analysis was performed twice, once by clamping each score and once without clamping. That is, instead of estimating separate parameters for the loading of each MB score on the latent factor, we also tested model fit when ‘clamping’ the parameters so that each MB score has the same loading for both time-measurements. The model with clamping showed slightly better fit to the data (RMSE = .087, BIC = 139.24) compared to the one without clamping (RMSE = .126, BIC = 144.96), and was therefore used in this analysis.

### Data availability

Open-Science Framework (OSF) project including: (1) a Matlab code for simulating RL and DDM-RL models (2) a .csv data file with empirical observations (fully anonymized) and (3) an R code that generates the internal consistency plots for both MB scores (choice & RT), and for simulated and observed data, can be found here: https://osf.io/zc24g/?view_only=d7f00134186c411986cc4de46b38edc5

## Supporting information

S1 Text Temporal stability analysis with a shorter time-gap (6-month). (DOCX)

S2 Text List of Members of the NSPN consortium. (PDF)

S1 Table Internal and temporal reliability scores for mean stay probability, main effect of reward and main effect of transition on stay probability for first-stage choices. (DOCX)

S2 Table Descriptive statistics for RL and DDM-RL hierarchical model parameters. (DOCX)

S3 Table Temporal stability estimates for hierarchical model parameters. (DOCX)

S1 Fig Predictions regarding the interaction effect of previous transition and reward on the probability that a learner will repeat the choice at the first stage (pStay) for a model-free (panel A) and model-based (panel B) learners. A model-free learner is assumed to be influenced by previous reward alone (if the previous trial was rewarded, the model-free learner is more likely to make the same choice at the first stage). For a pure model-based agent, previous reward should have the same effect in common transitions. However, in uncommon transitions, the chances of repeating the first stage choice is reduced when the previous trial was rewarded. MB-I_(choice)_ is the interaction which is zero for a pure model-free and positive for a model-based learner.
(TIF)

S2 Fig Histograms for baseline and follow-up measurements for both hierarchical MB scores. (**A**) MB-I_(choice)_, indicated the interaction score for the effect of previous reward and transition on the probability of sticking with the same first stage choice. (**B**) MB-II_(RT)_ indicated the differences in second stage RTs between uncommon and common trials. Positive values for both scores were assumed to indicate a higher involvement of model-based processing. The histograms suggest most of the population tend to show positive value in both scores.
(TIF)

S3 Fig Test-retest reliability for MB latent factor. In this model, each MB latent factor is predicting two observed MB scores (MB-I & II), separately for baseline (left side) and follow-up (right side). Estimates represent standardized beta coefficients. ***p < .001.
(TIF)

S4 Fig Q-Q plots for simulated vs. empirical RTs. To assess the ability of the DDM-RL to predict participants RTs, we calculated for each empirical RT_2_ distribution at baseline/follow-up nine RT percentiles (.1 to .9). We then simulated for each individual 50 experiments with 1000 trials each, based on the fitted parameters, and calculated RT percentiles from simulated data. The plot suggests a good match between empirical and predicted RTs as can be seen by a linear trend between the empirical vs. simulated percentiles (with a tendency of the model to overestimate long RTs). Each color representing a different individual.
(TIF)

S5 Fig Model predictions and empirical behaviour for the task’s second-stage, as a function of value discriminability. Here, we simulated for each participants Q-values using the individual’s parameters (hierarchical RL-DDM) and the sequence of events the individual experienced (i.e., rewards and transitions during performance). For each trial we calculated ΔQ-value (maximum-minimum), and averaged model predictions for choices and RTs (based on simulations of 100 decisions per trial). Therefore, for each trial we obtained empirical choices and RTs taken form participants behaviour as well as averaged choices and RTs simulated by the model, based on the trial-by-trial Q-values. Trials were then binned into five bins according to ΔQ-value of 0 to .2, .2 to .4, .4 to .6, .6 to .8 or .8 to 1 (represented in the x-axis across all plots). Results are presented separately for model-free and model-based participants (grouped by means of median split over the w-parameter estimates). (**A/B**) Probability of selecting the bandit with the higher Q-value, as a function of value discriminability (difference between high and low Q-value bandit). (**C/D**) Mean reaction-times as a function of value discriminability. Overall, these plots present a good fit between model prediction and participants’ behaviour, with no visual difference between model-based and model-free behaviour. Error bars for empirical data represent standard error.
(TIF)

S6 Fig Model predictions and empirical behaviour for the task’s second-stage RT quantiles, as a function of value discriminability. For each trial we obtained empirical choices and RTs taken form participants behaviour as well as averaged choices and RTs simulated by the model, based on the calculated trial-by-trial Q-values (see S5 Fig caption for further details). Trials were binned into five bins according to ΔQ-value of 0 to .2, .2 to .4, .4 to .6, .6 to .8 or .8 to 1 (represented in the x-axis across all plots). We then further binned for each individual RTs into five bins, separately for each ΔQ-value bin (total of 25 bins pre individual). We then calculated the mean RT (in seconds) for each of the 25 bins separately for model-free and model-based participants (group by means of median split over the w-parameter estimates). (A/B) Observed second-stage RTs as a function for model-free and model-based individuals. (C/D) Model predictions for model-free and model-based individuals. Overall, we did not find any differences between model-free and model-based individuals in terms of how good the model predicted RTs. This is despite a slight tendency of the model to predict quicker RTs for the last bin, and slower RTs for the fast bin. Error bars for empirical data represent standard error.
(TIF)

## References

[1] Dolan RJ, Dayan P. Goals and habits in the brain. Neuron. 2013;80: 312–325. 10.1016/j.neuron.2013.09.007 24139036 PMC3807793

[2] Wunderlich K, Smittenaar P, Dolan RJ. Dopamine enhances model-based over model-free choice behavior. Neuron. 2012;75: 418–424. 10.1016/j.neuron.2012.03.042 22884326 PMC3417237

[3] Don HJ, Goldwater MB, Otto AR, Livesey EJ. Rule abstraction, model-based choice, and cognitive reflection. Psychon Bull Rev. 2016;23: 1615–1623. 10.3758/s13423-016-1012-y 26907600

[4] Decker JH, Otto AR, Daw ND, Hartley CA. From creatures of habit to goal-directed learners: Tracking the developmental emergence of model-based reinforcement learning. Psychol Sci. 2016;27: 848–858. 10.1177/0956797616639301 27084852 PMC4899156

[5] Redgrave P, Rodriguez M, Smith Y, Rodriguez-Oroz MC, Lehericy S, Bergman H, et al Goal-directed and habitual control in the basal ganglia: implications for Parkinson’s disease. Nat Rev Neurosci. 2010;11: 760–772. 10.1038/nrn2915 20944662 PMC3124757

[6] Lee SW, Shimojo S, O’Doherty JP. Neural computations underlying arbitration between model-based and model-free learning. Neuron. 2014;81: 687–699. 10.1016/j.neuron.2013.11.028 24507199 PMC3968946

[7] Huang VS, Haith A, Mazzoni P, Krakauer JW. Rethinking motor learning and savings in adaptation paradigms: Model-free memory for successful actions combines with internal models. Neuron. 2011;70: 787–801. 10.1016/j.neuron.2011.04.012 21609832 PMC3134523

[8] Seger CA. Corticostriatal foundations of habits. Curr Opin Behav Sci. 2018;20: 153–160. 10.1016/j.cobeha.2018.01.006

[9] Beierholm UR, Anen C, Quartz S, Bossaerts P. Separate encoding of model-based and model-free valuations in the human brain. NeuroImage. 2011;58: 955–962. 10.1016/j.neuroimage.2011.06.071 21757014

[10] Gremel CM, Costa RM. Orbitofrontal and striatal circuits dynamically encode the shift between goal-directed and habitual actions. Nat Commun. 2013;4: 2264 10.1038/ncomms3264 23921250 PMC4026062

[11] Wunderlich K, Dayan P, Dolan RJ. Mapping value based planning and extensively trained choice in the human brain. Nat Neurosci. 2012;15: 786–791. 10.1038/nn.3068 22406551 PMC3378641

[12] Montague PR, Dolan RJ, Friston KJ, Dayan P. Computational psychiatry. Trends Cogn Sci. 2012;16: 72–80. 10.1016/j.tics.2011.11.018 22177032 PMC3556822

[13] Gillan CM, Kosinski M, Whelan R, Phelps EA, Daw ND. Characterizing a psychiatric symptom dimension related to deficits in goal-directed control. eLife. 2016;5 10.7554/eLife.11305 26928075 PMC4786435

[14] Voon V, Reiter A, Sebold M, Groman S. Model-based control in dimensional psychiatry. Biol Psychiatry. 2017;82: 391–400. 10.1016/j.biopsych.2017.04.006 28599832

[15] Vandaele Y, Janak PH. Defining the place of habit in substance use disorders. Prog Neuropsychopharmacol Biol Psychiatry. 2017; 10.1016/j.pnpbp.2017.06.029 28663112 PMC5748018

[16] Rangel A. Regulation of dietary choice by the decision-making circuitry. Nat Neurosci. 2013;16: 1717–1724. 10.1038/nn.3561 24270272 PMC4053793

[17] Cushman F. Action, outcome, and value: a dual-system framework for morality. Personal Soc Psychol Rev. 2013;17: 273–292. 10.1177/1088868313495594 23861355

[18] Daw ND, Gershman SJ, Seymour B, Dayan P, Dolan RJ. Model-based influences on humans’ choices and striatal prediction errors. Neuron. 2011;69: 1204–1215. 10.1016/j.neuron.2011.02.027 21435563 PMC3077926

[19] Dezfouli A, Balleine BW. Actions, action sequences and habits: evidence that goal-directed and habitual action control are hierarchically organized. PLoS Comput Biol. 2013;9: e1003364 10.1371/journal.pcbi.1003364 24339762 PMC3854489

[20] Doll BB, Duncan KD, Simon DA, Shohamy D, Daw ND. Model-based choices involve prospective neural activity. Nat Neurosci. 2015;18: 767–772. 10.1038/nn.3981 25799041 PMC4414826

[21] Kool W, Cushman FA, Gershman SJ. When does model-based control pay off? PLoS Comput Biol. 2016;12 10.1371/journal.pcbi.1005090 27564094 PMC5001643

[22] Otto AR, Gershman SJ, Markman AB, Daw ND. The curse of planning: dissecting multiple reinforcement-learning systems by taxing the central executive. Psychol Sci. 2013;24: 751–761. 10.1177/0956797612463080 23558545 PMC3843765

[23] Gillan CM, Otto AR, Phelps EA, Daw ND. Model-based learning protects against forming habits. Cogn Affect Behav Neurosci. 2015;15: 523–536. 10.3758/s13415-015-0347-6 25801925 PMC4526597

[24] Miller KJ, Botvinick MM, Brody CD. Dorsal hippocampus contributes to model-based planning. Nat Neurosci. 2017;20: 1269–1276. 10.1038/nn.4613 28758995 PMC5575950

[25] Gold JI, Shadlen MN. The neural basis of decision making. Annu Rev Neurosci. 2007;30: 535–574. 10.1146/annurev.neuro.29.051605.113038 17600525

[26] Ratcliff R. Parameter variability and distributional assumptions in the diffusion model. Psychol Rev. 2013;120: 281–292. 10.1037/a0030775 23148742 PMC3975928

[27] Ballard IC, McClure SM. Joint modeling of reaction times and choice improves parameter identifiability in reinforcement learning models. bioRxiv. 2018; 306720 10.1101/306720 PMC8930195 30664916

[28] Kiddle B, Inkster B, Prabhu G, Moutoussis M, Whitaker KJ, Bullmore ET, et al Cohort Profile: The NSPN 2400 Cohort: a developmental sample supporting the Wellcome Trust NeuroScience in Psychiatry Network. Int J Epidemiol. 2018;47: 18–19g. 10.1093/ije/dyx117 29177462 PMC5837633

[29] Culbreth AJ, Westbrook A, Daw ND, Botvinick M, Barch DM. Reduced model-based decision-making in schizophrenia. J Abnorm Psychol. 2016;125: 777–787. 10.1037/abn0000164 27175984 PMC4980177

[30] Deserno L, Huys QJM, Boehme R, Buchert R, Heinze H-J, Grace AA, et al Ventral striatal dopamine reflects behavioral and neural signatures of model-based control during sequential decision making. Proc Natl Acad Sci U S A. 2015;112: 1595–1600. 10.1073/pnas.1417219112 25605941 PMC4321318

[31] Rummery GA, Niranjan M. On-line Q-learning using connectionist systems. 1994.

[32] Sutton RS, Barto AG. Reinforcement learning: An introduction. MIT press; 1998.

[33] Krajbich I, Bartling B, Hare T, Fehr E. Rethinking fast and slow based on a critique of reaction-time reverse inference. Nat Commun. 2015;6: 7455 10.1038/ncomms8455 26135809 PMC4500827

[34] Pedersen ML, Frank MJ, Biele G. The drift diffusion model as the choice rule in reinforcement learning. Psychon Bull Rev. 2017;24: 1234–1251. 10.3758/s13423-016-1199-y 27966103 PMC5487295

[35] Frank MJ, Gagne C, Nyhus E, Masters S, Wiecki TV, Cavanagh JF, et al fMRI and EEG predictors of dynamic decision parameters during human reinforcement learning. J Neurosci. 2015;35: 485–494. 10.1523/JNEUROSCI.2036-14.2015 25589744 PMC4293405

[36] Luzardo A, Alonso E, Mondragón E. A Rescorla-Wagner drift-diffusion model of conditioning and timing. PLOS Comput Biol. 2017;13: e1005796 10.1371/journal.pcbi.1005796 29095819 PMC5685643

[37] Millner AJ, Gershman SJ, Nock MK, den Ouden HEM. Pavlovian control of escape and avoidance. J Cogn Neurosci. 2017;30: 1379–1390. 10.1162/jocn_a_01224 29244641

[38] Navarro DJ, Fuss IG. Fast and accurate calculations for first-passage times in Wiener diffusion models. J Math Psychol. 2009;53: 222–230. 10.1016/j.jmp.2009.02.003

[39] Tuerlinckx F, Maris E, Ratcliff R, De Boeck P. A comparison of four methods for simulating the diffusion process. Behav Res Methods Instrum Comput. 2001;33: 443–456. 10.3758/BF03195402 11816447

[40] Blurton SP, Kesselmeier M, Gondan M. Fast and accurate calculations for cumulative first-passage time distributions in Wiener diffusion models. J Math Psychol. 2012;56: 470–475. 10.1016/j.jmp.2012.09.002

[41] Gershman S. Reinforcement learning and drift-diffusion modeling, GitHub [Internet]. 2018. Available: https://github.com/sjgershm/RL_DDM

[42] Ratcliff R, McKoon G. The diffusion decision model: Theory and data for two-choice decision tasks. Neural Comput. 2008;20: 873–922. 10.1162/neco.2008.12-06-420 18085991 PMC2474742

[43] Wilkinson GN, Rogers CE. Symbolic description of factorial models for analysis of variance. J R Stat Soc Ser C Appl Stat. 1973;22: 392–399. 10.2307/2346786

[44] Schwarz G. Estimating the dimension of a model. Ann Stat. 1978;6: 461–464. 10.1214/aos/1176344136

[45] Green SB, Yang Y, Alt M, Brinkley S, Gray S, Hogan T, et al Use of internal consistency coefficients for estimating reliability of experimental task scores. Psychon Bull Rev. 2016;23: 750–763. 10.3758/s13423-015-0968-3 26546100 PMC5484005

[46] Lord FM, Novick MR, Birnbaum A. Statistical theories of mental test scores. Oxford, England: Addison-Wesley; 1968.

[47] Thompson BL, Green SB, Yang Y. Assessment of the maximal split-half coefficient to estimate reliability. Educ Psychol Meas. 2010;70: 232–251. 10.1177/0013164409355688

[48] Cronbach LJ, Furby L. How we should measure change: Or should we?., 74(1), 68. Psychol Bull. 1970;74: 68–80.

[49] Silva CF da, Hare TA. A note on the analysis of two-stage task results: How changes in task structure affect what model-free and model-based strategies predict about the effects of reward and transition on the stay probability. PLoS ONE. 2018;13: e0195328 10.1371/journal.pone.0195328 29614130 PMC5882146

[50] Kaplan D. Structural Equation Modeling: Foundations and extensions. SAGE Publications; 2008.

[51] Yang Y, Green SB. Coefficient alpha: a reliability coefficient for the 21st century? J Psychoeduc Assess. 2011;29: 377–392. 10.1177/0734282911406668

[52] Van Der Maas HLJ, Kan K-J, Marsman M, Stevenson CE. Network models for cognitive development and intelligence. J Intell. 2017;5: 16 10.3390/jintelligence5020016 PMC6526461 31162407

[53] Monsell S. Task switching. Trends Cogn Sci. 2003;7: 134–140. 10.1016/S1364-6613(03)00028-7 12639695

[54] Huys QJM, Maia TV, Paulus MP. Computational psychiatry: From mechanistic insights to the development of new treatments. Biol Psychiatry Cogn Neurosci Neuroimaging. 2016;1: 382–385. 10.1016/j.bpsc.2016.08.001 29560868

[55] Hauser TU, Will G-J, Dubois M, Dolan RJ. Annual Research Review: Developmental computational psychiatry. J Child Psychol Psychiatry. 0. 10.1111/jcpp.12964 30252127

[56] Huys QJM, Cools R, Gölzer M, Friedel E, Heinz A, Dolan RJ, et al Disentangling the roles of approach, activation and valence in instrumental and pavlovian responding. PLoS Comput Biol. 2011;7 10.1371/journal.pcbi.1002028 21556131 PMC3080848

