# Supplementary material for: Improving the reliability of model-based decision-making estimates in the two-stage decision task with reaction-times and drift-diffusion modeling
Source: PLoS Comput Biol. 2019 Feb 13;15(2):e1006803. doi: 10.1371/journal.pcbi.1006803 (PMC6391008; doi:10.1371/journal.pcbi.1006803)
Supplement: S1 Text — (DOCX) [file pcbi.1006803.s001.docx]

**S1 Text. Temporal stability analysis with a shorter time-gap (6-month).**

A subset of participants was re-tested between baseline and follow up measurements, ~6 months after the baseline measurement. We made use of this data to explore temporal stability for MB scores in a closer time proximity to the one reported in the main text with a larger sample.

**Participant exclusion and pre-processing.**

61 participants had data for both baseline and 6-month follow-up (female=33, male=28; mean age at baseline 18.94, range 14.41 to 24.37; mean time difference between the two time points was 6.53 months, range=5.04 to 8.04 months). We repeated the same pre-processing stages as reported in the main text, yet no participants had to be excluded for responding with the same key on more than 95% of the trials or for having implausible RTs (below 150_ms_) on more than 10% of the trials. For the remaining two-stage task data, the first trial in each block, as well as trials with implausible RTs (below 150_ms_) were omitted from the analysis (1.4% of the overall trials). The task at 6 month follow-up was exactly the same as described in the main text and had the same amount of trials as baseline (121 trials).

**Results.**

We repeated the same temporal analysis reported in the main text with a 6-month follow-up sample (N=61). For individual MB scores we found a Pearson correlation of r=-.05 (CI_95%_: -.30/.20) for MB-I_(choice)_ and r=.33 (CI_95%_: .08/.54) for MB-II_(RT)_. For hierarchical scores we found a Pearson correlation of r=.48 (CI_95%_: .26/.65) for MB-I_(choice)_ and r=.34 (CI_95%_: .10/.54) for MB-II_(RT)_. Therefore, overall temporal stability was low and very much in line with the reliability estimates we found with a larger sample, with ~18 month apart. The use hierarchical regression scores seemed to greatly improve the reliability for MB score I, but not II.
