# Supplementary material for: Improving the reliability of model-based decision-making estimates in the two-stage decision task with reaction-times and drift-diffusion modeling
Source: PLoS Comput Biol. 2019 Feb 13;15(2):e1006803. doi: 10.1371/journal.pcbi.1006803 (PMC6391008; doi:10.1371/journal.pcbi.1006803)
Supplement: S2 Text — (PDF) [file pcbi.1006803.s002.pdf]

Neuroscience in Psychiatry Network (NSPN) Consortium author list

Principal investigators:

Edward Bullmore (CI from 01/01/2017)

Raymond Dolan

Ian Goodyer (CI until 01/01/2017)

Peter Fonagy

Peter Jones

NSPN (funded) staff:

Michael Moutoussis

Tobias Hauser

Sharon Neufeld

Rafael Romero-Garcia

Michelle St Clair

Petra Vértes

Kirstie Whitaker

Becky Inkster

Gita Prabhu

Cinly Ooi

Umar Toseeb

Barry Widmer

Junaid Bhatti

Laura Villis

Ayesha Alrumaithi

Sarah Birt

Aislinn Bowler

Kalia Cleridou

Hina Dadabhoy

Emma Davies

Ashlyn Firkins

Sian Granville

Elizabeth Harding

Alexandra Hopkins

Daniel Isaacs

Janchai King

Danae Kokorikou

Christina Maurice

Cleo McIntosh

Jessica Memarzia

Harriet Mills

Ciara O'Donnell

Sara Pantaleone

Jenny Scott

Affiliated scientists:

Pasco Fearon

John Suckling

Anne-Laura van Harmelen

Rogier Kievit
