# Supplementary material for: Improving the reliability of model-based decision-making estimates in the two-stage decision task with reaction-times and drift-diffusion modeling
Source: PLoS Comput Biol. 2019 Feb 13;15(2):e1006803. doi: 10.1371/journal.pcbi.1006803 (PMC6391008; doi:10.1371/journal.pcbi.1006803)
Supplement: S1 Table — (DOCX) [file pcbi.1006803.s003.docx]

| S1 Table. Reliability scores for mean stay probability and main effects of previous transition and reward on stay probability. | | | |
| --- | --- | --- | --- |
|  |  | **CI_95%_** | |
|  | **Pearson r** | **lower** | **upper** |
| **Internal consistency (with 201 trials, only follow-up)** | | | |
| Mean stay probability | .96 | .95 | .97 |
| Effect of previous reward on stay probability | .87 | .85 | .89 |
| Effect of previous transition on stay probability | .93 | .92 | .95 |
| **Temporal stability estimates** | | | |
| Mean stay probability | .49 | .42 | .56 |
| Effect of previous reward on stay probability | .23 | .15 | .31 |
| Effect of previous transition on stay probability | .11 | .04 | .20 |
