# Supplementary material for: Improving the reliability of model-based decision-making estimates in the two-stage decision task with reaction-times and drift-diffusion modeling
Source: PLoS Comput Biol. 2019 Feb 13;15(2):e1006803. doi: 10.1371/journal.pcbi.1006803 (PMC6391008; doi:10.1371/journal.pcbi.1006803)
Supplement: S2 Table — (DOCX) [file pcbi.1006803.s004.docx]

| *S2 Table.* Descriptive statistics for RL and DDM-RL hierarchical model parameters | | | | | |
| --- | --- | --- | --- | --- | --- |
|  |  | mean | max | min | sd |
| RL model  (choice) | α_1_ | 0.29 | 0.92 | 0.02 | 0.18 |
|  | α_2_ | 0.37 | 0.87 | 0.02 | 0.19 |
|  | *w* | 0.44 | 0.74 | 0.22 | 0.10 |
|  | λ | 0.54 | 0.90 | 0.21 | 0.12 |
|  | *p* | 0.10 | 0.26 | -0.03 | 0.05 |
|  | β_1_ | 6.58 | 15.27 | 2.70 | 2.29 |
|  | β_2_ | 3.79 | 8.38 | 1.65 | 1.19 |
|  |  |  |  |  |  |
| DDM-RL model  (choice & RT) | α_1_ | 0.22 | 0.87 | 0.00 | 0.17 |
|  | α_2_ | 0.35 | 0.91 | 0.02 | 0.19 |
|  | *w* | 0.42 | 0.74 | 0.15 | 0.09 |
|  | λ | 0.54 | 0.89 | 0.22 | 0.11 |
|  | *p* | 0.14 | 0.33 | -0.05 | 0.08 |
|  | *b*_1_ | 4.16 | 6.81 | 2.26 | 0.88 |
|  | *a*_1_ | 1.40 | 2.53 | 0.87 | 0.22 |
|  | τ_1_ | 0.26 | 0.46 | 0.14 | 0.05 |
|  | *b*_2_ | 2.63 | 5.99 | 1.25 | 0.79 |
|  | *a*_2_ | 1.45 | 1.93 | 0.90 | 0.15 |
|  | τ_2_ | 0.28 | 0.50 | 0.12 | 0.06 |
