# Supplementary material for: Improving the reliability of model-based decision-making estimates in the two-stage decision task with reaction-times and drift-diffusion modeling
Source: PLoS Comput Biol. 2019 Feb 13;15(2):e1006803. doi: 10.1371/journal.pcbi.1006803 (PMC6391008; doi:10.1371/journal.pcbi.1006803)
Supplement: S3 Table — (DOCX) [file pcbi.1006803.s005.docx]

| *S3 Table.* Temporal stability estimates for hierarchical model parameters | |
| --- | --- |
|  | Spearman’s correlation |
| **RL model (choice only)** |  |
| α_1_ | .15 _[.07-.23]_ |
| α_2_ | .39 _[.32-.46]_ |
| *p* | .32 _[.25-.40]_ |
| β_1_ | .26 _[.28-.43]_ |
| β_2_ | .27 _[19-.35]_ |
| λ | .19 _[.11-.27]_ |
|  |  |
| **RL-DDM (choice & RT)** |  |
| α_1_ | .19 _[.11-.27]_ |
| α_2_ | .35 _[.28-.42]_ |
| *p* | .41 _[.34-.48]_ |
| λ | .13 _[.05-.21]_ |
| *b*_1_ | .22 _[.14-.30]_ |
| *b*_2_ | .21 _[.13-.29]_ |
| *a*_1_ | .39 _[.31-.45]_ |
| *a*_2_ | .36 _[.28-.42]_ |
| τ_1_ | .43 _[.36-.50]_ |
| τ_2_ | .37 _[.30-.44]_ |
| *Note.* Estimates in brackets represent 95% confidence intervals. | |
