# Supplementary figures and images for: Improving the reliability of model-based decision-making estimates in the two-stage decision task with reaction-times and drift-diffusion modeling
Source: PLoS Comput Biol. 2019 Feb 13;15(2):e1006803. doi: 10.1371/journal.pcbi.1006803 (PMC6391008; doi:10.1371/journal.pcbi.1006803)

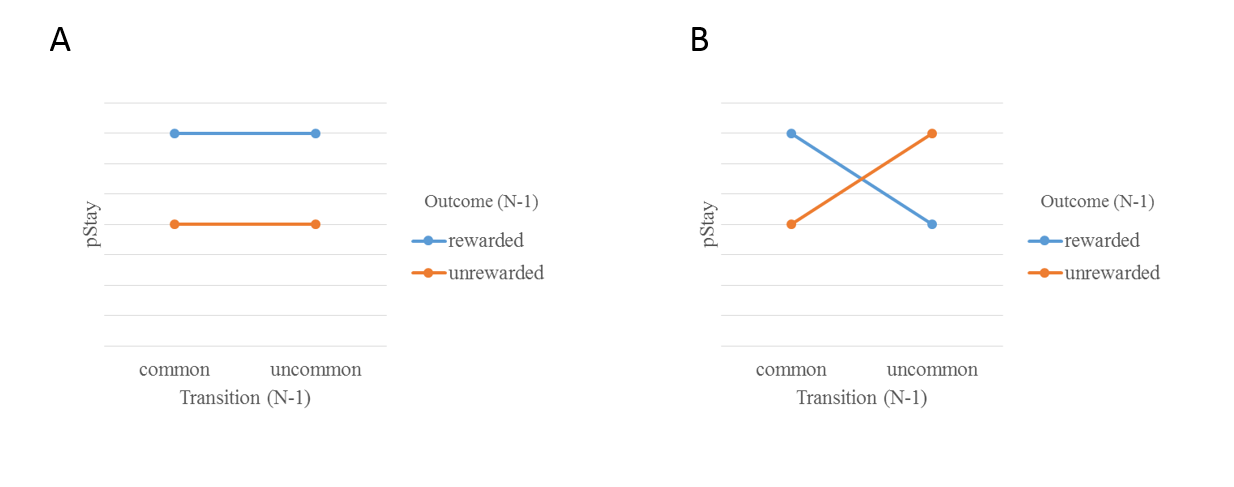

Supplement: S1 Fig — Predictions regarding the interaction effect of previous transition and reward on the probability that a learner will repeat the choice at the first stage (pStay) for a model-free (panel A) and model-based (panel B) learners. A model-free learner is assumed to be influenced by previous reward alone (if the previous trial was rewarded, the model-free learner is more likely to make the same choice at the first stage). For a pure model-based agent, previous reward should have the same effect in common transitions. However, in uncommon transitions, the chances of repeating the first stage choice is reduced when the previous trial was rewarded. MB-I(choice) is the interaction which is zero for a pure model-free and positive for a model-based learner. (TIF) [file pcbi.1006803.s006.tif]

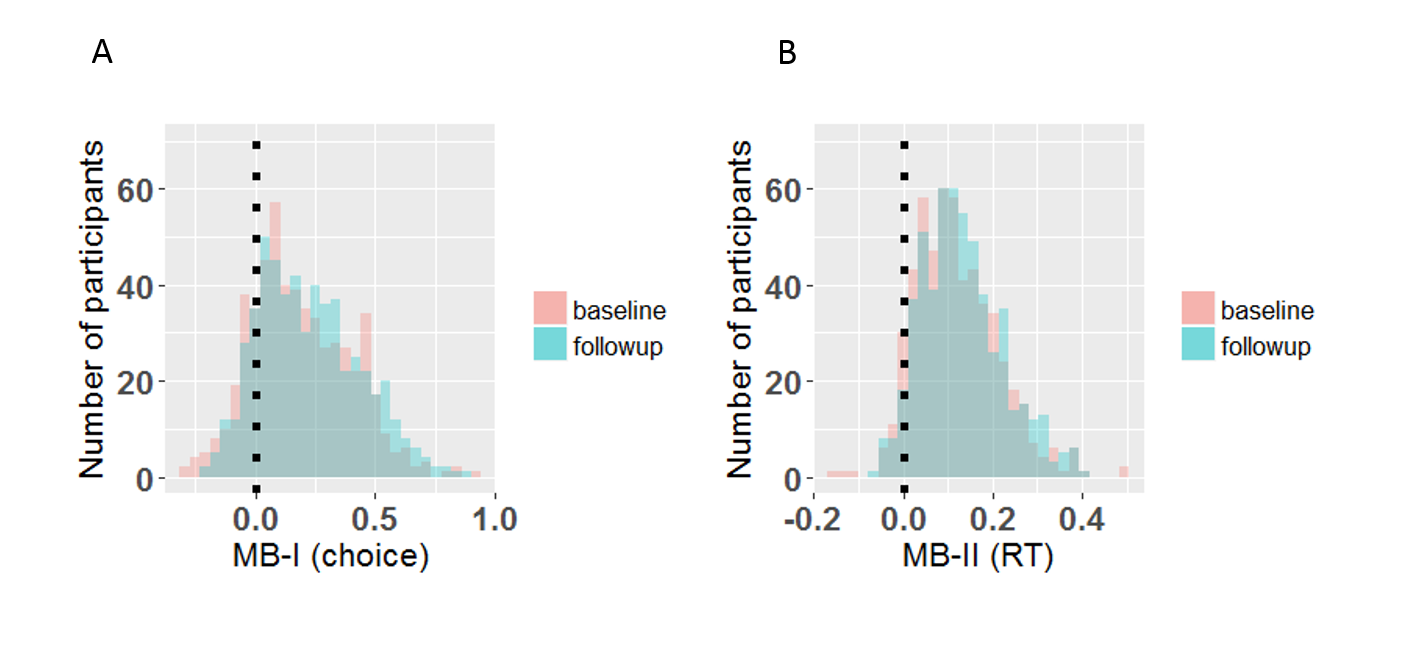

Supplement: S2 Fig — (A) MB-I(choice), indicated the interaction score for the effect of previous reward and transition on the probability of sticking with the same first stage choice. (B) MB-II(RT) indicated the differences in second stage RTs between uncommon and common trials. Positive values for both scores were assumed to indicate a higher involvement of model-based processing. The histograms suggest most of the population tend to show positive value in both scores. (TIF) [file pcbi.1006803.s007.tif]

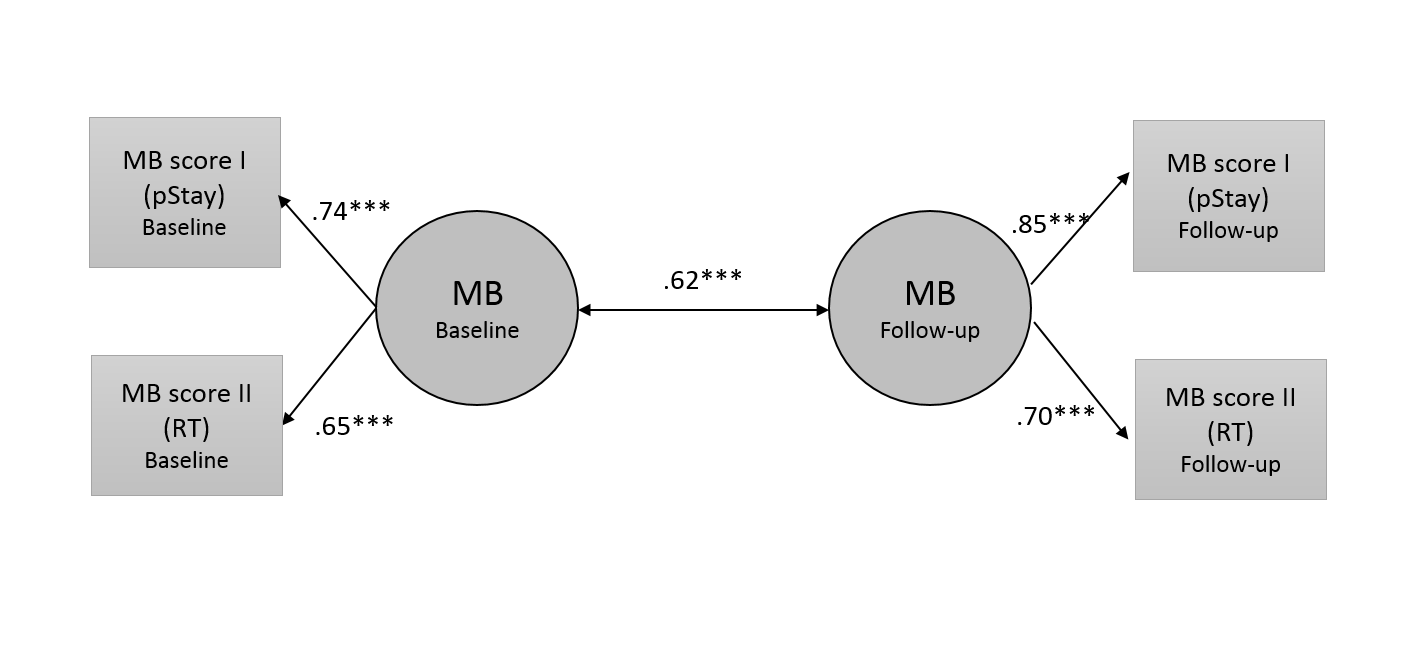

Supplement: S3 Fig — In this model, each MB latent factor is predicting two observed MB scores (MB-I & II), separately for baseline (left side) and follow-up (right side). Estimates represent standardized beta coefficients. ***p < .001. (TIF) [file pcbi.1006803.s008.tif]

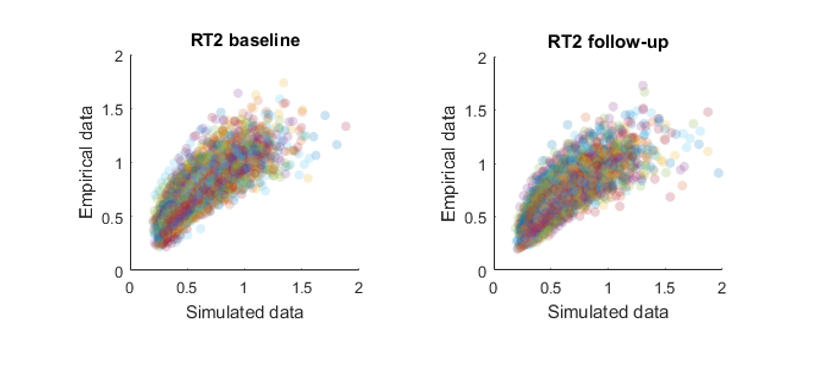

Supplement: S4 Fig — To assess the ability of the DDM-RL to predict participants RTs, we calculated for each empirical RT2 distribution at baseline/follow-up nine RT percentiles (.1 to .9). We then simulated for each individual 50 experiments with 1000 trials each, based on the fitted parameters, and calculated RT percentiles from simulated data. The plot suggests a good match between empirical and predicted RTs as can be seen by a linear trend between the empirical vs. simulated percentiles (with a tendency of the model to overestimate long RTs). Each color representing a different individual. (TIF) [file pcbi.1006803.s009.tif]

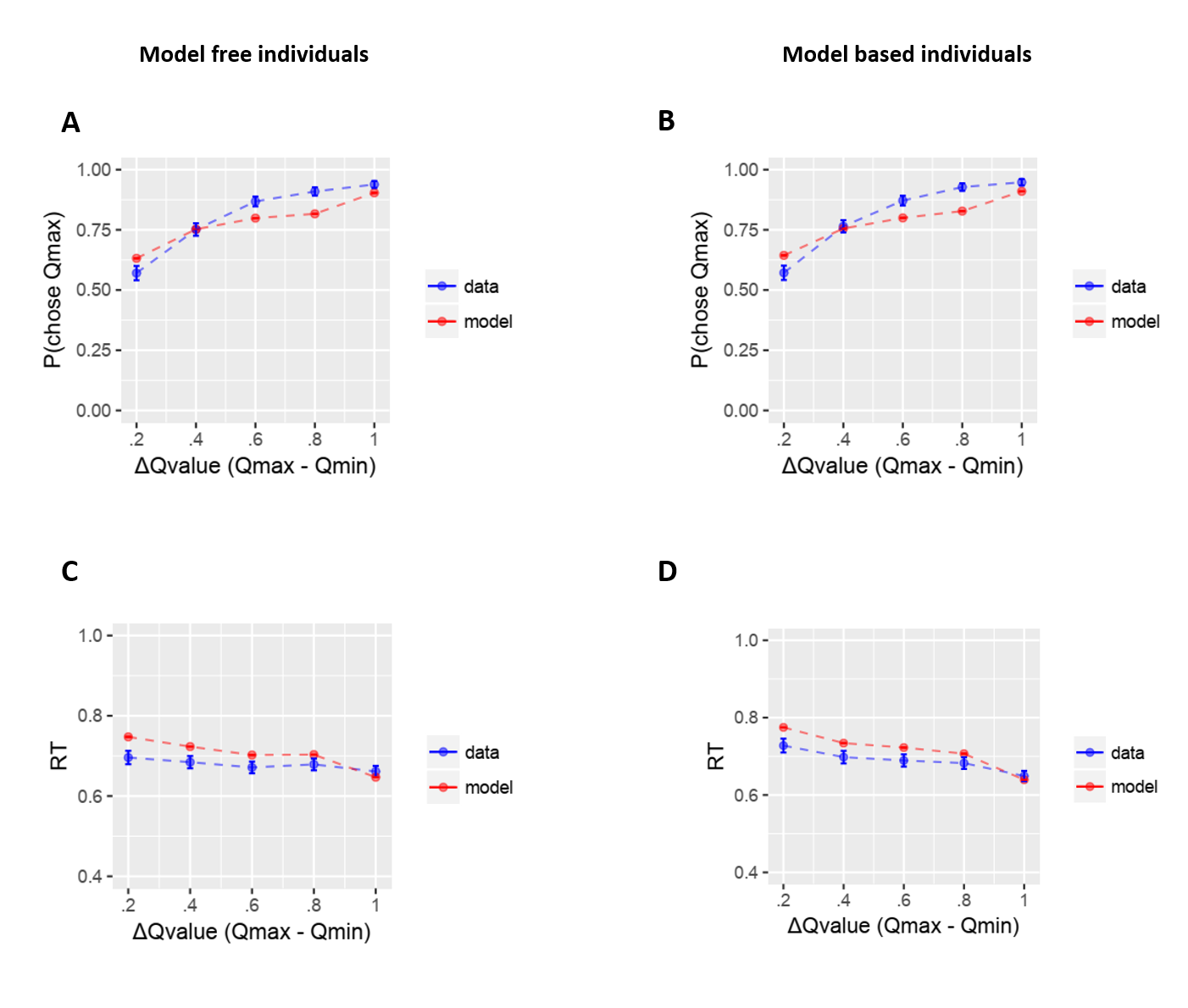

Supplement: S5 Fig — Here, we simulated for each participants Q-values using the individual’s parameters (hierarchical RL-DDM) and the sequence of events the individual experienced (i.e., rewards and transitions during performance). For each trial we calculated ΔQ-value (maximum-minimum), and averaged model predictions for choices and RTs (based on simulations of 100 decisions per trial). Therefore, for each trial we obtained empirical choices and RTs taken form participants behaviour as well as averaged choices and RTs simulated by the model, based on the trial-by-trial Q-values. Trials were then binned into five bins according to ΔQ-value of 0 to .2, .2 to .4, .4 to .6, .6 to .8 or .8 to 1 (represented in the x-axis across all plots). Results are presented separately for model-free and model-based participants (grouped by means of median split over the w-parameter estimates). (A/B) Probability of selecting the bandit with the higher Q-value, as a function of value discriminability (difference between high and low Q-value bandit). (C/D) Mean reaction-times as a function of value discriminability. Overall, these plots present a good fit between model prediction and participants’ behaviour, with no visual difference between model-based and model-free behaviour. Error bars for empirical data represent standard error. (TIF) [file pcbi.1006803.s010.tif]

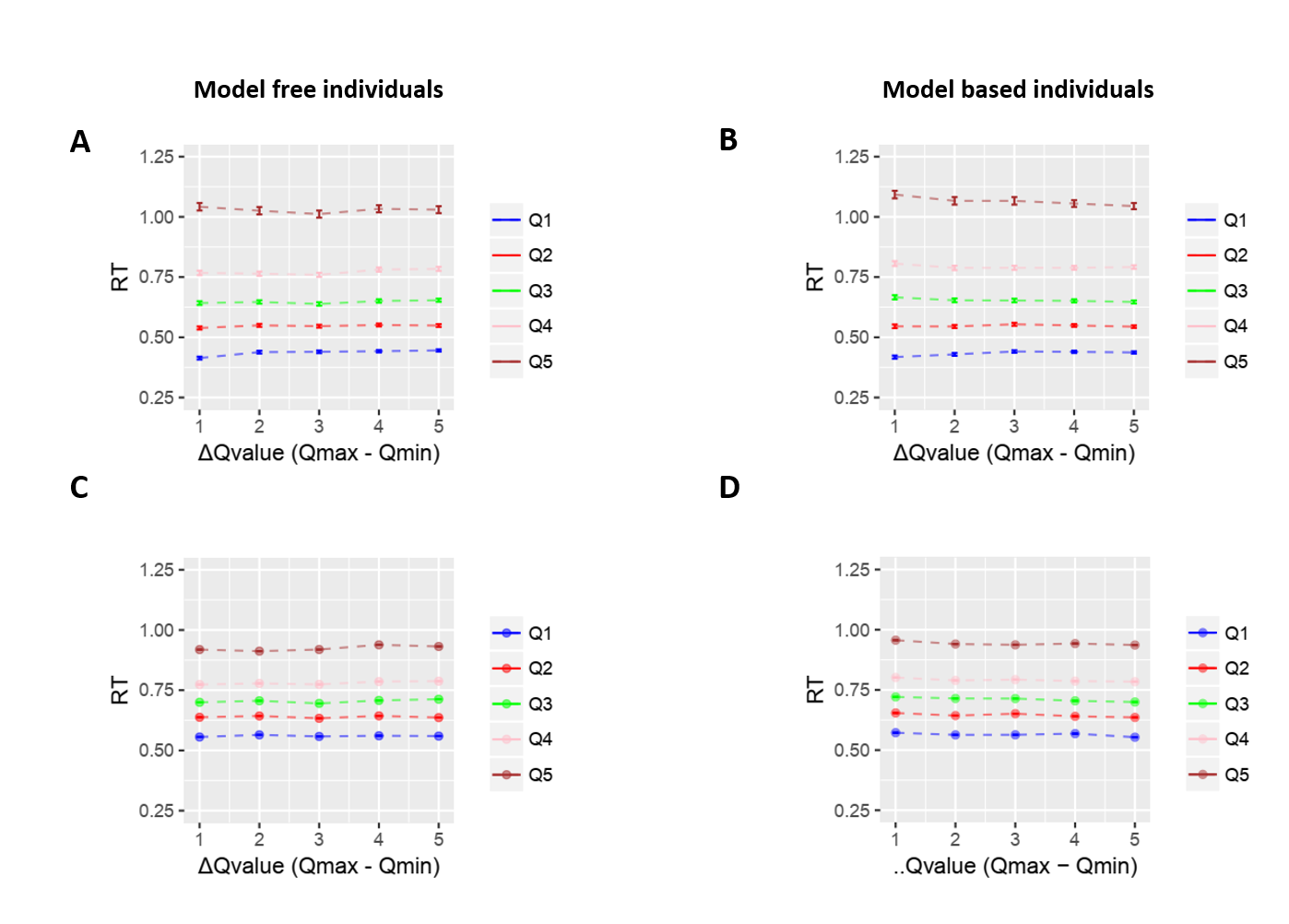

Supplement: S6 Fig — For each trial we obtained empirical choices and RTs taken form participants behaviour as well as averaged choices and RTs simulated by the model, based on the calculated trial-by-trial Q-values (see S5 Fig caption for further details). Trials were binned into five bins according to ΔQ-value of 0 to .2, .2 to .4, .4 to .6, .6 to .8 or .8 to 1 (represented in the x-axis across all plots). We then further binned for each individual RTs into five bins, separately for each ΔQ-value bin (total of 25 bins pre individual). We then calculated the mean RT (in seconds) for each of the 25 bins separately for model-free and model-based participants (group by means of median split over the w-parameter estimates). (A/B) Observed second-stage RTs as a function for model-free and model-based individuals. (C/D) Model predictions for model-free and model-based individuals. Overall, we did not find any differences between model-free and model-based individuals in terms of how good the model predicted RTs. This is despite a slight tendency of the model to predict quicker RTs for the last bin, and slower RTs for the fast bin. Error bars for empirical data represent standard error. (TIF) [file pcbi.1006803.s011.tif]
